# Conservation and Diversification of tRNA t^6^A-Modifying Enzymes across the Three Domains of Life

**DOI:** 10.3390/ijms232113600

**Published:** 2022-11-06

**Authors:** Chenchen Su, Mengqi Jin, Wenhua Zhang

**Affiliations:** School of Life Sciences, Lanzhou University, 222 South Tianshui Road, Lanzhou 730030, China

**Keywords:** tRNA modification, *N*^6^-threonylcarbamoyladenosine synthetases, TsaC/Sua5, TsaD/Kae1/Qri7, KEOPS, TsaD–TsaB–TsaE, protein–protein interactions, tRNA binding, ATPase activity, structure–function relationship, human disease

## Abstract

The universal *N*^6^-threonylcarbamoyladenosine (t^6^A) modification occurs at position 37 of tRNAs that decipher codons starting with adenosine. Mechanistically, t^6^A stabilizes structural configurations of the anticodon stem loop, promotes anticodon–codon pairing and safeguards the translational fidelity. The biosynthesis of tRNA t^6^A is co-catalyzed by two universally conserved protein families of TsaC/Sua5 (COG0009) and TsaD/Kae1/Qri7 (COG0533). Enzymatically, TsaC/Sua5 protein utilizes the substrates of *L*-threonine, HCO_3_^−^/CO_2_ and ATP to synthesize an intermediate *L*-threonylcarbamoyladenylate, of which the threonylcarbamoyl-moiety is subsequently transferred onto the A37 of substrate tRNAs by the TsaD–TsaB –TsaE complex in bacteria or by the KEOPS complex in archaea and eukaryotic cytoplasm, whereas Qri7/OSGEPL1 protein functions on its own in mitochondria. Depletion of tRNA t^6^A interferes with protein homeostasis and gravely affects the life of unicellular organisms and the fitness of higher eukaryotes. Pathogenic mutations of YRDC, OSGEPL1 and KEOPS are implicated in a number of human mitochondrial and neurological diseases, including autosomal recessive Galloway–Mowat syndrome. The molecular mechanisms underscoring both the biosynthesis and cellular roles of tRNA t^6^A are presently not well elucidated. This review summarizes current mechanistic understandings of the catalysis, regulation and disease implications of tRNA t^6^A-biosynthetic machineries of three kingdoms of life, with a special focus on delineating the structure–function relationship from perspectives of conservation and diversity.

## 1. Introduction

**Figure 1:**
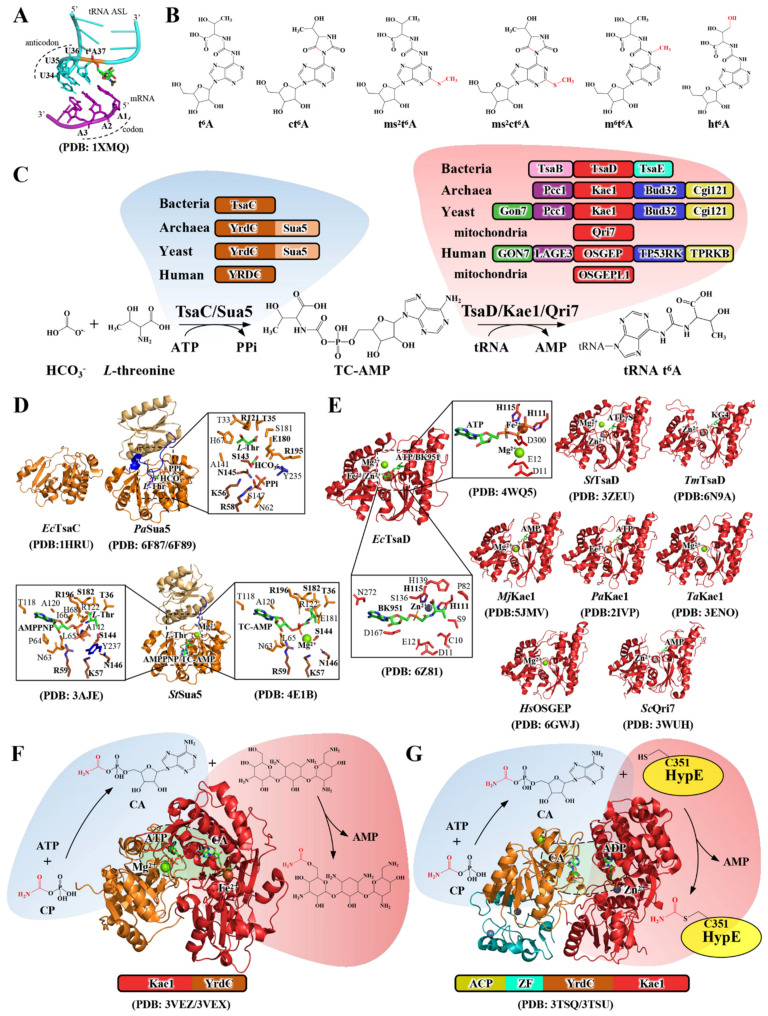
Structure, function and biosynthetic pathway of tRNA t^6^A. (**A**). The crystal structure of t^6^A-ASL of *E coli.* tRNA^Lys^ (UUU) paired with mRNA in ribosomes (omitted for clarity) [22]. (**B**). Chemical structures of t^6^A and t^6^A derivatives with modifications highlighted in red. t^6^A, *N^6^*-threonylcarbamoyladenosine; ct^6^A, cyclic *N^6^*-threonylcarbamoyladenosine; ms^2^t^6^A, 2-methylthio-*N^6^*-threonylcarbamoyladenosine; ms^2^ct^6^A, 2-methylthiocyclic *N^6^*-threonylcarbamoyladenosine; m^6^t^6^A, *N^6^*-methyl-*N^6^*-threonylcarbamoyladenosine; ht^6^A, hydroxy-*N^6^*-threonylcarbamoyladenosine. (**C**). Enzymatically validated and representative tRNA t^6^A-modifying enzymes in the three domains of life and the enzymatic pathway of tRNA t^6^A biosynthesis. The catalytic members of TsaC/Sua5 and TsaD/Kae1/Qri7 families are colored in brown and red, respectively. Evolutionarily related proteins are color-coded. (**D**). Crystal structures of *E. coli* TsaC [31], *P. abyssi* Sua5 [32] and *S. tokudaii* Sua5 [33,34]. The YrdC domain and Sua5 domain are displayed in brown and light yellow, respectively, and the connecting loop between the two domains is marked in blue. The bound ligand (*L*-threonine, HCO_3_^−^ and PPi in *P. abyssi* Sua5, AMPPNP, *L*-threonine, TC-AMP and Mg^2+^ in *S. tokodaii* Sua5) are shown in detail and the KxR···SxN tetrad motifs are indicated. (**E**). Crystal structures of TsaD/Kae1/Qri7 proteins from different species (*E. coli* [35,36], *S. typhimurium* [37], *T. maritima* [38], *M. jannaschii* [39], *P. abyssi* [40], *T. acidophilum* [41], *H. sapiens* [42] and *S. cerevisiae* [43]). The metal-binding motif (HxxH···D) and nucleotide-binding site are indicated in each structure. KG4, carboxyadenosine. (**F**,**G**). Crystal structure, domain organization and enzymatic catalysis of *S. tenebrarius* TobZ [34] (**F**) and *E. coli* HypF [44]. (**G**). TobZ comprises an N-terminal Kae1 domain (red) and a C-terminal YrdC domain (brown); HypF comprises an N-terminal ACP domain (greenish), a ZF domain (cyan), a YrdC domain (brown) and a C-terminal Kae1 domain (red). The chemical transformation in the reactions catalyzed by the YrdC and Kae1 domains is shown in TobZ (**F**,**G**). The reaction channels between the two domains are marked in light green. CP, carbamoyl phosphate; CA, carbamoyladenylate. PDB codes are given in brackets.

The transfer RNAs (tRNAs), which cooperate with ribosomes to translate genetic codes of the messenger RNA (mRNA), contain a large number of chemically distinct post-transcriptional modifications (https://iimcb.genesilico.pl/modomics, accessed on 1 October 2020) [1,2,3,4]. These modifications at different positions are needed for maintaining the chemical stability, stabilizing the tertiary structure, decoding ability and the decay of tRNAs [5,6,7,8,9,10]. The wobble position 34 and position 37 (3′ adjacent to anticodon) are two hotspots that install the largest diversity of chemical modifications [3,4,11,12,13,14]. The *N*^6^-threonylcarbamoyladenosine (t^6^A), which denotes the incorporation of an *L*-threonine via a ureido linkage at the N6 nitrogen of adenosine (Figure 1A,B) [15,16], is universally found at position 37 of ANN-decoding (N being A, U, C and G) tRNAs from the three domains of life (Table 1) [11,12,14,17,18]. Structurally, t^6^A extends its planar ring via intramolecular hydrogen bonds and π–π stacking interaction with its 5′-adjacent base U36 and prevents the intra-loop Watson–Crick pairing between U33 and A37 (Figure 1A) [14,19,20,21]. The t^6^A-stabilized conformation of anticodon stem loop (ASL) facilitates the entry of amino-acylated tRNAs into the ribosomal A-site, wherein t^6^A principally promotes the formation of a codon–anticodon duplex via forming extra hydrogen bonds and overcoming the low enthalpy of binding for U–A base pairs [5,22,23,24,25]. Thus, tRNA t^6^A modification plays a crucial role in safeguarding the translational fidelity by means of enhancing the correct recognition of the start codon (AUG) and preventing frameshift as well [7,26,27,28]. In addition, t^6^A promotes efficient aminoacylation of cognate tRNAs [17,27,29,30].

In addition, tRNA t^6^A is the substrate of a variety of t^6^A hypermodifications that include cyclic *N^6^*-threonylcarbamoyladenosine (ct^6^A) [45,46], hydroxy-*N*^6^-threonylcarbamoyladenosine (ht^6^A) [28], 2-methylthio-*N*^6^-threonylcarbamoyladenosine (ms^2^t^6^A) [47], *N*^6^-methyl-*N*^6^-threonylcarbamoyladenosine (m^6^t^6^A) [48] and 2-methylthiocyclic *N*^6^-threonylcarbamoyladenosine (ms^2^ct^6^A) (Figure 1B) [49]. Collectively, t^6^A and t^6^A-derived nucleosides at position 37 of tRNAs contribute to the translational efficiency and cellular proteostasis regulation [7,23,26,50]. The loss or hypomodification of tRNA t^6^A leads to the death of unicellular organisms, the development of higher eukaryotes [50,51] and a number of human diseases (Table 1) [52,53,54,55], including mitochondrial respiration defects [29], neurological disorders [42,56] and diabetes [57,58].

Biosynthesis of tRNA t^6^A requires two universally conserved protein families of TsaC/Sua5 (COG0009) [59] and TsaD/Kae1/Qri7 (COG0533) [60], which belong to the minimal gene-sets and the last universal common ancestors (LUCA) [61,62,63,64]. Comparative genomics and proteomic analyses have identified a complete ensemble of enzymes capable of catalyzing tRNA t^6^A biosynthesis in the three domains of life (Table 1 and Figure 1C) [65]. As conventionally represented, tRNA t^6^A biosynthesis in bacteria requires TsaC (YrdC), TsaD (YgjD), TsaB (YeaZ) and TsaE (YjeE) [66,67]; in archaea and eukaryotic cytosol, it requires a Sua5/YRDC and KEOPS complex that contains Kae1, Bud32, Cgi121, Pcc1 and Gon7 [68,69,70,71,72]; in eukaryotic mitochondria, it requires Sua5/YRDC and Qri7/OSGEPL1, which are nuclear-encoded and imported into mitochondria [29,60,61,73,74]. The enzymatic synthesis of tRNA t^6^A takes place in two steps (Figure 1C): in the first step, TsaC/Sua5 protein independently catalyzes the condensation of *L*-threonine, HCO_3_^−^/CO_2_ and ATP to form an intermediate Threonylcarbamoyl-adenylate (TC-AMP) and an inorganic pyrophosphate (PPi) [32,67,75,76]; in the second step, the TC-moiety is catalytically transferred onto the N6 nitrogen of tRNA A37 by TsaD/Kae1/Qri7 protein with support of a varying number and types of accessory proteins in bacteria, archaea and eukaryotes [65,66,68,69,70,71,72,73,74]. The molecular mechanisms of tRNA t^6^A-modifying enzymes vary among different biological systems. This review work is focused on analyzing the structure–function relationship and portrays an overview of conservation and diversification pertaining to molecular workings of these tRNA t^6^A biosynthetic machineries in the three domains of life.

**Table 1:** Summary of the biosynthetic enzymes of tRNA t^6^A and other related tRNA t^6^A hypomodified nucleosides found and validated in different organisms. Abbreviations: TsaDBE, TsaD–TsaB–TsaE; YdiECB, YdiE (TsaD)-YdiC (TsaB)-YdiB (TsaE); ^CT^, cytosol; ^MT^, mitochondria; N.D., not determined.

Modified	Organisms	Enzymes	Functions and Deficit-Associated Phenotypes	Refs
t^6^A	Bacteria	*E. coli* TsaC and TsaDBE *B. subtilis*YwlCandYdiECB	tRNA t^6^A is required for recognizing the start codon (AUG) and prevents frameshift in translation; the depletion of *tsaC* and *tsaD* leads to cell death.	[17,60,66,67]
	Archaea	*P. abyssi/M. jannaschi/H. volcanii* Sua5 and KEOPS	*H. volcanii* Pcc1 (KEOPS subunit) is not essential to cellular life; the deletion of *pcc1* reduces the level of t^6^A modification and leads to decreased growth rate.	[39,71,77]
	Eukarya	*S. cerevisiae* Sua5 and KEOPS^(CT)^	Deletion of *sua5* and KEOPS (*kae1*, *bud32*, *cgi121 and pcc1*) abolishes the tRNA t^6^A formation and leads to pleiotropic lethal phenotypes.	[41,65,68,70]
		*S. cerevisiae* Sua5 and Qri7^(MT)^	Qri7 is essential for yeast life. Deletion of *qri7* leads to mitochondrial genome instability and abnormal morphology.	[18,30,73,78]
		*H. sapiens* YRDC and KEOPS^(CT)^	Deletion or mutations of *YRDC* and KEOPS (*OSGEP*, *TP53RK*, *TPRKB*, *LAGE3 and GON7*) leads to depletion of tRNA t^6^A and is implicated in Galloway–Mowat syndrome.	[42,56,79,80]
		*H. sapiens* YRDC and OSGEPL1^(MT)^	Deletion of *YRDC* and *OSGEPL1* causes mitochondrial dysfunction (interferes with protein synthesis and respiratory activities) and is clinically associated with an onset of myoclonus epilepsy with ragged-red fibers (MERRF).	[29,74]
ct^6^A	Bacteria	*E. coli* TcdA/ *B. subtilis* YrvM	Bacterial ct^6^A-tRNA^Lys^ (UUU) is involved in promoting decoding efficiency.	[46,48]
	Eukarya	*S. cerevisiae* TCD1 and TCD2	Yeast ct^6^A-tRNAs are required for yeast respiratory growth under nonfermenting conditions.	[46]
		*A. thaliana* enzymes N.D.	N.D.	[49]
ms^2^t^6^A	Bacteria	*B. subtilis* YqeV (MtaB)	*B. subtilis* ms^2^t^6^A-tRNA^Lys^ (UUU) is required for the accurate translation of AAG codon.	[58]
	Eukarya	*T. brucei* MtaB	Depletion of ms^2^t^6^A confers no significant growth.	[49]
		*A. thaliana* enzymes N.D.	N.D.	[49]
		*H. sapiens* CDKAL1	ms^2^t^6^A-tRNA^Lys^ (UUU) is required for the accurate translation of AAA codon and its deficit is associated with type 2 diabetes.	[58]
ms^2^ct^6^A	Bacteria	*B. subtilis* YrvM and YqeV	Depletion of ms^2^ct^6^A confers no significant growth or temperature-sensitivity phenotypes.	[49]
	Eukarya	*T. brucei* TcdA and MtaB	ms^2^ct^6^A-tRNA promotes decoding efficiency and contributes to cell growth in inhibitory conditions or under stress.	[49]
		*A. thaliana* enzymes N.D.	N.D.	[49]
m^6^t^6^A	Bacteria	*E. coli* TrmO	m^6^t^6^A enhances the decoding ability of tRNA^Thr^ and discriminates tRNAs^Thr^ for ACY codons from other isoacceptors.	[48]
	Eukarya	*R. norvegicus* TrmO	m^6^t^6^A-tRNA^Ser^ (GCU) is responsible for the AGY codons.	[48]
		*H. sapiens* TRMO	N.D.	[48]
ht^6^A	Eukarya	*Echinoderm* mitochondria t^6^A enzymes using the substrate of Hydroxy-*L*-threonine	*M. nudus* mt-tRNA^Lys^ (UUU) is employed to decodes asparagine instead of lysine.	[28]

## 2. The Biochemical and Structural Aspects of tRNA t^6^A Catalysis

### 2.1. Structure–Function Relationship of TsaC/Sua5 in TC-AMP Biosynthesis

Two homologs of TsaC/Sua5 family are found in almost all sequenced organisms (Figure 1C) [59,81]. While TsaC and YRDC are single domain proteins comprising ~200 amino acids, Sua5 consists of an N-terminal YrdC/TsaC domain and an extra Sua5 domain (~100 amino acids), which are connected by a 40 amino acid-long segment (Figure 1D). The phylogenetic distribution shows that, with few exceptions, the single-domain TsaC and YRDC are present in bacteria and higher eukaryotes, while Sua5 is found in archaea and yeast (Figure 1C) [81]. The lethal phenotype of *E. coli tsaC* deletion and loss of tRNA t^6^A could be rescued and restored by either *B. subtilis ywlC* (*tsaC*) or *S. cerevisiae SUA5*, and vice versa [59,81,82], substantiating an independent catalytic role in TC-AMP synthesis.

*E. coli* TsaC folds into an α/β twisted open sheet structure with antiparallel adjacent β-strands in the center and α-helices flanking on the exterior side, forming a baseball glove-like structure. The structure of TsaC aligns well with the YrdC domain of Sua5, of which the Sua5 domain adopts a Rossmann fold with an inner β-sheet composed of five β-strands and framed by three α-helices [31,32,33,76]. The binding and coordination of TC-AMP substrates (ATP, *L*-threonine and HCO_3_^−^), TC-AMP and the byproduct PPi have been determined in the catalytic centers of TsaC/Sua5 proteins (Figure 1D and Table 2) [32,33,75,76]. The structure reveals that the interdomain segment wraps over the catalytic center and functions as an essential gating loop to regulate the catalytic activity of Sua5 (Figure 1D) [31,32]. The structure–function analysis provides a mechanistic basis underscoring the chemical transformations of *L*-threonine, HCO_3_^−^ and ATP en route to TC-AMP: 1) *L*-threonine is first bound to react non-enzymatically with HCO_3_^−^ in the closed catalytic pocket, generating an intermediate *N*-carboxy-*L*-threonine; 2) the subsequent opening of the gating loop permits the entry of ATP for adenylation with *N*-carboxy-*L*-threonine, of which the carboxyl group performs a nucleophilic attack, giving birth to TC-AMP and PPi [31,32,76].

### 2.2. Structure–Function Relationship of TsaD/Kae1/Qri7 in t^6^A Biosynthesis

Though many essential cellular roles have been associated with TsaD/Kae1/Qri7 family proteins [60,78,81,86], the central and convergent one is the catalytic function in tRNA t^6^A biosynthesis (Figure 1C). Crystal structures of TsaD/Kae1/OSGEP/Qri7 proteins exhibit a typical “acetate and sugar kinase/heat shock protein 70/actin” (ASKHA) superfamily characteristic of comprising two similar ancestral oligonucleotide-binding (OB) domains on either side of a large cleft with a canonical ATP-binding site at the bottom (Figure 1E), except for local variations incurred by insertion or deletion of connecting loops [35,37,38,40,41,42,43,73,87,88,89]. The overall structural architecture and configuration of the catalytic sites are conserved among TsaD/Kae1/OSGEP/Qri7 proteins. The binding and coordination of nucleotides (ATP, ADP and AMP) have been determined in crystal structures (Figure 1E) and verified in biochemical assays (Table 2). In addition, different divalent cations (Fe^2+^, Zn^2+^ and Mn^2+^) are bound in coordination with ATP or ADP in the catalytic sites [35,37,38,40,41,73,87,90]. The metal ions are octahedrally coordinated by a highly conserved HxxH···D motif and two water molecules and play a canonical role in coordinating the γ- and β-phosphate of ATP and stabilizing the structural conformation of loops on the edge of the α-helices (Figure 1E) [35,37,40,41,91]. Biochemical assays exhibited differentiated effects of metal ions on stimulating the t^6^A-catalytic activities of TsaD/Kae1/Qri7 proteins, demonstrating that TsaD/Kae1/Qri7 proteins are versatile in making the best use of these metal cations [65,74].

However, TsaD/Kae1/OSGEP/Qri7 does not necessarily consume ATP throughout the chemical transformation in tRNA t^6^A catalysis. The ATP-binding site must be used for binding TC-AMP or A37 of tRNA (Figure 1C). As expected, crystal structures revealed that KG4 (analog of carboxy-AMP) and BK951 (analog of TC-AMP) are bound in the same ATP-binding sites of TsaD [36,38]. In addition, the bound TC-AMP analogs adopt a similar anti-conformation as that seen for ATP and ADP bound in catalytic sites of TsaD/Kae1/Qri7 proteins (Figure 1E). Thus, the ATP-binding site is, in fact, used for TC-AMP binding and is involved in catalyzing the transfer of TC-moiety. The structure revealed that TC-moiety is bound to the active site Zn^2+^ atom via an oxygen atom from both the phosphonate and the carboxylate moieties, as the binding of A37 of tRNA in the catalytic sites of TsaD/Kae1/Qri7 proteins is presently unknown. Functional analysis combined with the computational modeling of the A37 of tRNA suggested that TC-AMP and A37 could concomitantly accommodate in a manner that TC-moiety is bound to the metal ion and A37 is perpendicularly positioned to the TC-AMP plane. The catalysis exploits a putative metal oxyanion hole, in which the incoming N6 of tRNA A37 performs a nucleophilic attack at the carbamoyl bond of TC-AMP [36,38].

### 2.3. Evolutionary Implications of a Functional Cooperation between YrdC-like Domain and Kae1-like Domain

Interestingly, a YrdC-like domain and a Kae1-like domain are consecutively fused and functionally associated in *S. tenebrarius* TobZ [34] and *E. coli* HypF [44,92]. Crystal structures exhibit that TobZ consists of a Kae1 domain at the N-terminus and a YrdC domain at the C-terminus (Figure 1F) [34]. By contrast, HypF comprises an N-terminal acylphosphatase (ACP) domain, a Zn finger-like (ZF) domain, a YrdC domain and a C-terminal Kae1 domain (Figure 1G) [44,92]. The overall structures of both YrdC and Kae1 domains of TobZ and HypF are well matched to TsaC (Figure 1D) and Kae1 (Figure 1E), respectively [34,92]. Furthermore, configurations of the catalytic centers are conserved, including catalytic motifs in TsaC (KxR···SxN) and Kae1(HxxH···D) [31,34,40,92]. Although the organizing order of Kae1 domain and YrdC domain in TobZ and HypF is reversed, they cooperate to catalyze a two-step carbamoylation reaction: first, the YrdC domain catalyzes the formation of an intermediate carbamoyladenylate (CA) using substrates of carbamoyl phosphate (CP) and ATP; second, the Kae1 domains in TobZ and HypF catalyze the transfer of carbamoyl-moiety from CA onto tobramycin via an *O*-carbamoylation (Figure 1F) [34] and Cystine 351 of HypE via an *S*-carbamoylation (Figure 1G) [44,92], respectively. These two evolutionarily ancient carbamoylation reactions are analogous to the threonylcarbamoylation reaction in tRNA t^6^A biosynthesis (Figure 1C). The two types of reaction involve a classic adenylation transformation (R-COOH+ATP → R-C=O-AMP+PPi). In TobZ and HypF, the YrdC domain and Kae1 domain interact to create an electrostatic potential gradient channel for efficient transfer of CA (Figure 1F,G) [34,44,92]. In contrast, TsaC/Sua5 proteins are capable of catalyzing the formation of TC-AMP independent of the t^6^A-catalysis by TsaD/Kae1/Qri7, and vice versa [67,73]. However, TC-AMP is a chemically unstable intermediate, and it decomposes spontaneously into AMP and *L*-threonine isocyanate (*L*-Thr-NCO) in minutes under physiological conditions [29,32,67,73]. Therefore, it is tempting to hypothesize that TsaC/Sua5 and TsaD/Kae1/Qri7 proteins could interact to facilitate an efficient delivery of TC-AMP to the catalytic sites of TsaD/Kae1/Qri7 proteins.

## 3. The Diversification of tRNA t^6^A Biosynthetic Systems

Genetic and biochemical validations demonstrated that Qri7/OSGEPL1 forms a minimal tRNA t^6^A biosynthetic system in combination with Sua5/YRDC [73,74]. Whereas bacterial TsaD requires TsaB and TsaE to perform t^6^A-catalysis [38,66,67], archaea and eukaryote Kae1 catalyze the t^6^A biosynthesis in the form of a KEOPS complex [70,71,72,79]. It appears that TsaD/Kae1/OSGEP proteins, in comparison to Qri7/OSGEPL1 proteins, have evolved to adopt diversified and easy-to-tune mechanisms underlying the t^6^A-catalytic activation in coping with the increasing biological complexity.

### 3.1. The Dynamic TsaD–TsaB–TsaE Complex in Bacteria

Proteomic analyses revealed that TsaD is involved in an essential protein–protein interaction network with TsaB (a paralog of TsaD) and TsaE that is not evolutionarily related to any other tRNA t^6^A-modifying enzymes (Figure 1C) [35,38,63,84,93,94]. While neither *E. coli* TsaD–TsaB [35,65,66] nor *B. subtilis* TsaD–TsaB [67] exhibited any t^6^A-catalytic activity, *T. maritime* TsaD–TsaB showed basal catalytic activity in tRNA t^6^A biosynthesis [38]. Consistent with the in vivo tRNA t^6^A formation analyses, the t^6^A-catalytic function of TsaD–TsaB is fully activated by TsaE [35,38,65,66,67]. Genetic and biochemical analyses demonstrated that neither the lethal phenotype or depletion of tRNA t^6^A caused by deletion of *E. coli tsaD* could be complemented by *B. subtilis ydiE* (*tsaD)* or *S. cerevisiae KAE1*, manifesting that highly specific interactions play a critical role in molecular recognitions of TsaB and TsaE by TsaD [17,60,63,78,93].

**Figure 2:**
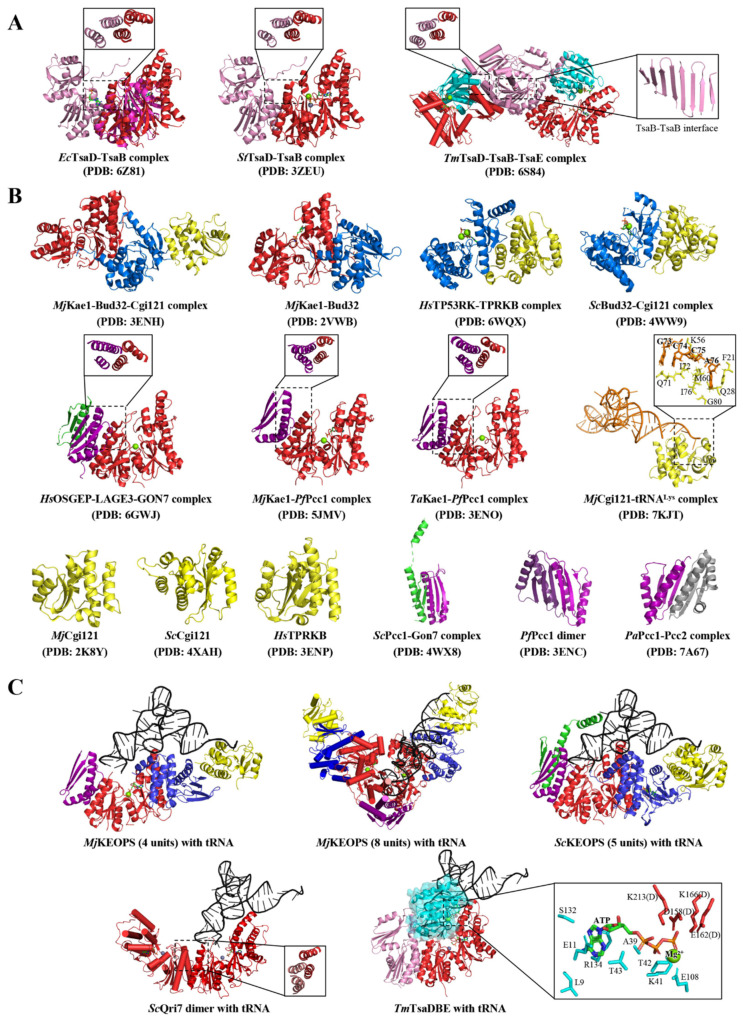
Structural assembly of tRNA t^6^A-modifying enzymes in the three domains of life. (**A**). Crystal structures of *Ec*TsaD–TsaB [36], *St*TsaD–TsaB [37] and *Tm*TsaD–TsaB–TsaE [87]. TsaD, TsaB and TsaE are colored in red, pink and cyan, respectively. In *Ec*TsaD–TsaB, an equivalent TsaB is structurally aligned to TsaD. The conserved “helical bundle” at the interacting interface between TsaD and TsaB is shown in the inserts. TsaB–TsaB interacting interface in *Tm*TsaD–TsaB–TsaE are shown in the insert. (**B**). Crystal structures of KEOPS subunits and subcomplexes from different species (*Mj*Kae1–Bud32–Cgi121 [41], *Mj*Kae1–Bud32 [99], *Hs*TP53RK–TPRKB [100], *Sc*Bud32–Cgi121 [101], *Hs*OSGEP–LAGE3–GON7 [42], *Mj*Kae1–*Pf*Pcc1 [39], *Ta*Kae1–*Pf*Pcc1 [41], *Mj*Cgi121–tRNA^Lys^ [72], *Mj*Cgi121 [41], *Sc*Cgi121 [101], *Hs*TPRKB [41], *Sc*Pcc1–Gon7 [101], *Pf*Pcc1 [41] and *Pa*Pcc1–Pcc2. Kae1, Bud32, Cgi121, Pcc1, Gon7 and their homologous proteins are shown in red, blue, yellow, purple and green, respectively. The conserved helical bundles at the Kae1/OSGEP–Pcc1/LAGE3 binding interface are shown in inserts. The tRNA^Lys^ 3′ CCA-coordinating residues of *Mj*Cgi121 are shown in detail. (**C**). Structural model of *Mj*KEOPS in complex with tRNA was generated based on the crystal structure of *Mj*Cgi121–tRNA [72]. The structural model of 8-subunit *Mj*KEOPS or 5-subunit *Sc*KEOPS in complex with tRNA was built by structural alignment using *Mj*KEOPS–tRNA as superposing references; models of *Sc*Qri7 dimer or TsaD–TsaB–TsaE in complex with tRNA were built by structural alignment using Kae1-tRNA as reference coordinates. The conserved “helical bundle” at the *Sc*Qri7 dimerization interface is shown in the insert. As depicted in TsaD–TsaB–TsaE–tRNA model, bindings of TsaE and tRNA are mutually exclusive. The ATP-binding residues at the *Tm*TsaE–TsaD interface are shown in detail. PDB codes are given in brackets. Abbreviations: *Ec*, *E. coli*; *St*, *S. typhimurium*; *Tm*, *T. maritima*; *Mj*, *M. jannaschii*; *Hs*, *H. sapiens*; *Sc*, *S. cerevisiae*; *Ta*, *T. acidophilum*; *Pf*, *P. furiosus*; *Pa*, *P. abyssi*.

TsaB is concomitantly present with TsaD in all bacterial genomes but has no close orthologs in eukaryotes [81,93]. Structures reveal that TsaB also belongs to the ASKHA superfamily and adopts a canonical two-lobed HSP70/actin-like fold, each of which comprises a five-stranded mixed β-sheet surrounded by three α-helices [95,96]. While the N- lobe could be superimposed onto that of TsaD (Figure 2A), the truncated C-lobe is less conserved than that of TsaD and does not form a similar OB fold in support of binding nucleotides and metal ions [35,37,87]. TsaD interacts with TsaB via a conserved “helical bundle” interacting interface that is created by two pairs of N-terminal α-helices of TsaD and TsaB (Figure 2A) [35,37,87]. In addition, TsaD–TsaB dimer formation involves the C-terminal tail of TsaB, which extends over the interacting interface onto TsaD (Figure 2A). Furthermore, the hyperthermophilic *T. maritime* TsaD–TsaB dimer further oligomerizes into a tetramer depicted as TsaD–TsaB–TsaB–TsaD, which is mediated by an extension of the β-stranded sheet of the C-terminal domains of TsaB (Figure 2A) [85,87,97,98]. Structural comparison revealed that a conserved motif GPGXXTGXR located on the “helical bundle” region of TsaB is re-configured to create an atypical nucleotide-binding site, which is occupied by an ADP or AMP in the structures of an *E. coli* TsaD–TsaB complex [35,36].

TsaE folds into a structure comprising a seven-intertwining stranded mixed β-sheet with three connecting α-helices on either side [85,87,102]. TsaE is structurally related to the P-loop NTPase characterized by mono-nucleotide-binding fold that canonically catalyzes hydrolysis of the β-γ phosphate ester bond of the nucleotide [103,104]. The ATP-binding site is located at the C-edge of the parallel portion of the β-sheet and involves two canonical Walker motifs (A/B) and two Switch (I/II) loops between the central β-strand and the following α-helix [85,87,102]. The binding of ATP or ADP to TsaE has been observed in crystal structures of TsaE [87,102] and characterized in in vitro analyses (Table 2) [35,85]. In-solution small-angle X-ray scattering (SAXS), sedimentation velocity and gel filtration analyses confirmed that TsaE exists in an equilibrium of monomers and homodimers [35,84,103]. SAXS analysis showed that only the monomeric TsaE binds to the TsaD–TsaB heterodimer but not TsaD–TsaD or TsaB–TsaB homodimers in the presence of ATP [35,85]. The crystal structure of the *T. maritime* TsaD–TsaB–TsaE complex revealed that TsaE is bound at the TsaD–TsaB interface (Figure 2A) and ATP is bound at the interface of TsaE and TsaD with its γ-phosphate group being coordinated by side chains from both subunits (Figure 2C) [85,87]. Functional analyses demonstrated that a dynamic assembly of a TsaD–TsaB–TsaE complex is essential for tRNA t^6^A catalysis [35,85], during which TsaE plays a role in promoting the catalytic turnover via “re-setting” the catalytic conformation of TsaD [38].

### 3.2. The KEOPS Machinery in Archaea and Eukarya

Yeast Kae1 was previously identified to be part of the KEOPS complex (kinase, putative endopeptidase, and other proteins of small size) [68] or EKC (endopeptidase-like kinase chromatin-associated) [69], which consists of Kae1, Bud32, Cgi121, Pcc1 and Gon7. The evolutionarily conserved KEOPSs share a common core made up of Kae1, Bud32, Cgi121 and Pcc1 (Figure 1C) [27,41,68,69], with exceptions that yeast and human KEOPS contains an extra Gon7 and Drosophila KEOPS lacks Cgi121 [42,50,79,101]. KEOPS has been associated with a number of fundamental cellular processes, including tRNA t^6^A modification [27,39,65,70], telomere replication [41,55,67,68,105,106,107,108] and transcription activation [26,69,108]. Dysfunction of KEOPS complex abolishes the tRNA t^6^A formation in vivo [42,70,79], leads to shortened telomeres [41,109] and impairs protein synthesis in archaea and eukaryotes [56,110]. In humans, mutations of KEOPS genes are implicated in neurological disorders, including the Galloway–Mowat syndrome (GAMOS), which is characterized by early-onset steroid-resistant nephrotic syndrome combined with mesangial sclerosis microcephaly and brain anomalies [42,56,80,111].

**Figure 3:**
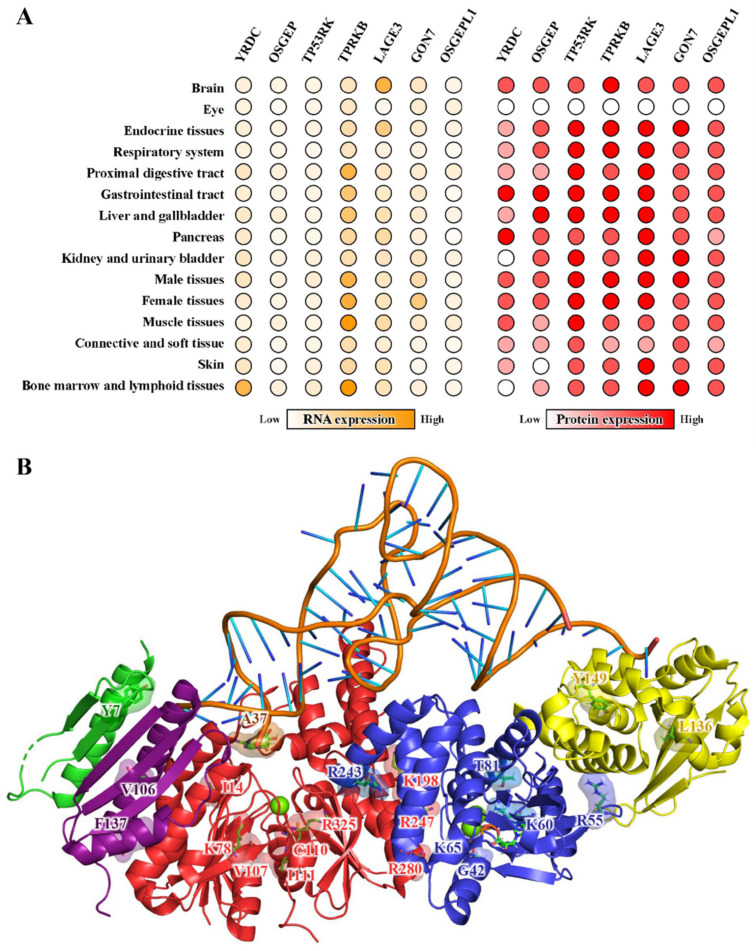
Expression patterns of human tRNA t^6^A-modifying enzymes and the pathogenic mutations of human KEOPS. (**A**). Transcriptomic and proteomic profiles of *YRDC*, *OSGEP*, *TP53RK*, *TPRKB*, *LAGE3*, *GON7* and *OSGEPL1* in different organs and tissues of humans (Human Protein Atlas) [114,115]. Values were scaled and normalized to the highest that are coded in orange and red, respectively. Blank circles denote not determined. (**B**). The structural model of human KEOPS in complex with tRNA. The model was generated by aligning the crystal structure of *M. jannaschii* Cgi121–tRNA (PDB: 7KJT) onto a composite structural model of human KEOPS built using *H. sapiens* OSGEP–LAGE3–GON7 (PDB: 6GWJ) and *H. sapiens* TP53RK–TPRKB (PDB: 6WQX). The represented mutations associated with Galloway–Mowat syndrome are projected structures of *osgep*, *tp53rk*, *tprkb*, *lage3* and *gon7*, which are colored in red, blue, yellow, purple and green, respectively.

Composite KEOPS models were built based on protein–protein interactions using crystal structures of KEOPS subcomplexes (Figure 2B) [39,41,42,72,100,101]. In the architecture of KEOPS, Kae1/OSGEP binds to Bud32/TP53RK, which folds into a bi-lobal configuration characteristic of eukaryotic protein kinase, including an ATPase-dependent kinase Rio2 [101,112,113]. C-lobe of Bud32/TP53RK protrudes into the catalytic center of Kae1/OSGEP [42,101]. On one end of Kae1/OSGEP–Bud32/TP53RK, the N-terminal domain of Kae1/OSGEP forms a “helical bundle” interacting interface with Pcc1/LAGE3 (Figure 2B), which further binds to Gon7/GON7 via forming an extended antiparallel β-sheet and contacts between α-helices; on the opposite end, the N-lobe of Bud32/TP53RK makes extensive contacts with Cgi121/TPRKB, which folds into a globular structure consisting of a four central antiparallel β-strands abutted by two α-helices on one side and five or six α-helices on the other [41,100,101]. Yeast or human KEOPS forms a pentameric complex that is linearly organized as Gon7–Pcc1–Kae1–Bud32–Cgi121 (Figure 2C and Figure 3B) whereas archaean KEOPS forms a V-shaped dimer of a tetrameric Pcc1–Kae1–Bud32–Cgi121 complex via the dimerization of Pcc1 (Figure 2C) [39,41,71,101].

The structure–function relationship and molecular workings of KEOPS have been described in detail in a recent review paper [116]. To put it simply, Kae1/OSGEP is the t^6^A catalytic subunit but remains catalytically inactive. It performs t^6^A catalysis in the form of a KEOPS complex. Bud32/TP53RK lacks structural motifs for being an atypical kinase but retains conserved elements required for ATP binding and hydrolysis. Bud32/TP53RK contributes to promoting the t^6^A-catalytic activity of KEOPS at the cost of consuming ATP and autophosphorylation that are positively regulated by Cgi121/TPRKB [88,89]. By doing so, Cgi121/TPRKB stimulates the t^6^A-catalytic activity of Kae1 in a positively cooperative manner [71,100]. Pcc1/LAGE3 folds into a KH-like domain that is frequently involved in RNA binding [41]. The fifth subunits Gon7 and GON7, which are found in yeast and humans, are intrinsically disordered proteins but acquire partial structure in the presence of Pcc1 and LAGE3, respectively [42,101]. The structured region of Gon7/GON7 folds into an antiparallel stranded β-sheet and two continuous α-helices. The connecting region between the second β-strand and first α-helix is disordered in the structures (Figure 2B) [42,101]. Gon7/GON7 interacts with Pcc1/LAGE3 via the same interacting interface of Pcc1 homodimer and inhibits the dimerization of Pcc1/LAGE3 in yeast and humans [42,101].

## 4. Molecular Interactions between tRNAs and tRNA t^6^A-Modifying Enzymes

Both in vivo and in vitro analyses on tRNA t^6^A formation demonstrated that not only the frequencies of t^6^A modification vary among tRNAs of the same origin but the t^6^A biosynthetic enzymes of different systems exhibit different catalytic activities on the same tRNAs (Table 3) [27,29,65,74]. It is assumed that the frequency of t^6^A modification of tRNAs is determined largely by the molecular interactions between substrate and enzymes. However, very little is known about how tRNA t^6^A-modifying enzymes discriminate ANN-decoding tRNAs from non-substrate tRNA and how the A37 of tRNA is docked into the t^6^A-catalytic site.

### 4.1. Sequence Motifs and Structural Determinants of t^6^A tRNA Substrate

Mature *L*-shaped ANN-decoding tRNAs of different origins could be t^6^A-modified by enzymes of different organisms (Table 3) [27,72,74]. These sets of cross-validation assays revealed a number of tRNA substrate properties pertaining to sequence and structural motifs: (1) U36A37A38 motif is a universal determinant for all t^6^A-modifying enzymes [27,30,117]; (2) C32 is an essential determinant for KEOPSs [27]; (3) the C10U11 motif of the D-stem is a determinant for archaean KEOPS [72]; (4) C33 is an anti-determinant for bacterial TsaD–TsaB–TsaE and yeast KEOPS [27]; (5) the 3′ CCA addition contributes to efficient t^6^A modification by KEOPS [72].

### 4.2. Structural Models of t^6^A-Modifying Enzymes in Complex with tRNA

Based on the only crystal structure of *M. jannaschii* Cgi121–tRNA^Lys^ (UUU) (Figure 2B) [72], composite structure models of four-subunit KEOPS–tRNA, eight-subunit KEOPS–tRNA, five-subunit KEOPS–tRNA, TsaD–TsaB–tRNA and Qri7–Qri7–tRNA are generated by means of structural alignment (Figure 2C and Figure 3B). Except for the Archaean KEOPS–tRNA model that has been validated by electron microscopy and mutagenesis studies [72], other models have not been experimentally validated and may incur potential inaccuracy. These models are used in this work to provide an architectural overview of tRNA in complex with t^6^A-catalytic machineries of different biological systems.

All these models manifest that the binding of the tRNA A37 to the catalytic centers of TsaD/Kae1/Qri7 proteins necessitates contributions of the conserved “helical bundle” interacting interfaces of TsaD–TsaB, Kae1/OSGEP–Pcc1/LAGE3 and Qri7–Qri7 (Figure 2). Mutations disrupting the interface abolished the t^6^A-catalytic activities of TsaD–TsaB–TsaE [35], KEOPS [39] and Qri7 [73]. Judged from the structures and models (Figure 2), the interface is not only essentially involved in the formation of the complexes built around TsaD/Kae1/Qri7 but may play a contributing role in docking ASL of tRNAs into catalytic centers of TsaD/Kae1/Qri7 proteins. Crystal structures *E. coli* TsaD–TsaB [35,36] and *T. maritima* TsaD–TsaB–TsaE [38] revealed that the formation of the “helical bundle” interfaces creates an atypical ADP/AMP-binding site, which might bind adenines of substrate tRNA. Mutational studies of *S. cerevisiae* Qri7 showed that only one molecule of tRNA is sterically allowed to be lodged in Qri7–Qri7 dimer (Figure 2C) [73]. In contrast, OSGEPL1 (the human counterpart of Qri7) functions as a monomer and does not form such a signature “helical bundle” interacting interface [74]. However, it was demonstrated that OSGEPL1 has evolved to make use of a post-translational modification mechanism (acylation of lysine residues) in regulating the t^6^A-catalytic activation [74].

### 4.3. Binding of tRNAs to TsaC/Sua5

Interestingly, high affinity interactions between tRNA and *E. coli* TsaC have been determined (Table 4) [31,59], though a direct binding of tRNAs to TsaC/Sua5 protein is dispensable in the in vitro reconstitution of tRNA t^6^A (Figure 1C) [67,73]. It is still not known whether such an intriguing binding phenomenon is involved in the *E. coli* tRNA t^6^A modification in a cellular context. Mechanistically, *E. coli* TsaC folds into an α/β twisted open-sheet structure with a large positively-charged surface surrounding the catalytic center, which favors the binding of nucleic acids, preferably double-stranded RNA (Figure 1D) [31]. Therefore, the binding of tRNA to *E. coli* TsaC is deemed promiscuous and is not related to tRNA t^6^A. Alternatively, tRNA mediates a transient interaction between TsaC and TsaD–TsaB in analogy to the interaction between YrdC domain and Kae1 domain in TobZ and HypF (Figure 1F,G) [34,92]. By doing so, the two catalytic centers are brought in proximity for efficient delivery of TC-AMP from TsaC to TsaD. More puzzlingly, the activity of Sua5 in hydrolyzing ATP into AMP and PPi is potentiated by the addition of tRNA [65], though no interaction between Sua5 and tRNA has been determined. Kinetic analysis on tRNA t^6^A formation also suggested that a direct binding of TsaC to TsaD in the presence of tRNA might have played a role in protecting the bound TC-AMP from decomposing into AMP and *L*-Thr-NCO via shielding it from water [36].

### 4.4. Interaction between tRNA and TsaD–TsaB–TsaE Complex

Structural characterization of the molecular interactions between tRNA and TsaD–TsaB–TsaE is scarce, apart from a set of quantitative binding analysis (Table 4). In-solution SAXS analysis confirmed that only one molecule of tRNA is bound to the dimer of the TsaD–TsaB heterodimer, forming an unsymmetrical complex depicted as tRNA–TsaD–TsaB–TsaB–TsaD [38]. The SAXS model of tRNA–TsaD–TsaB shows that tRNA occupies the binding region of TsaE in TsaD–TsaB and is therefore bound to TsaD–TsaB in a mutually exclusive manner to TsaE. Biochemical analysis confirmed that *T. maritima* TsaD–TsaB binds to *E. coli* tRNA^Thr^ with a *K_D_* value of 1.3 µM and the presence of *T. maritima* TsaE prevents the TsaD–TsaB complex from binding tRNA [85].

TsaE is an intrinsically weak ATPase whose activity is stimulated by a TsaD–TsaB dimer [35,84,85,118]. TsaE-catalyzed ATP hydrolysis is required for additional enzymatic turnover and occurs after the release of t^6^A-modified tRNA. Once the bound ATP is hydrolyzed, the ADP-bound TsaE is dissociated from TsaD–TsaB [35], creating an access and time window for binding of TC-AMP and tRNA in the active site of TsaD for the next catalytic cycle [38,85]. However, the ATPase-null TsaE mutant (mutation in the Walker B motif) is still capable of binding ATP and stimulating t^6^A catalytic activity of TsaD–TsaB in a comparable level as wild type TsaE [35,38]. The binding affinity between TsaD–TsaB and ATP-bound TsaE (*K_D_* = 0.34 µM) is tri-fold higher than that of TsaD–TsaB and tRNA (*K_D_* = 1.3 µM) [38,79,85,91]. It is therefore hypothesized that TsaE-catalyzed ATP hydrolysis plays a role in dissociating TsaE from TsaD–TsaB and in re-setting the active conformation of TsaD–TsaB [38]. Structure and function analysis demonstrated that TsaE works as a “molecular switch” triggered by ATP hydrolysis. However, the t^6^A-catalytic activity of TsaD–TsaB could be prohibitively stalled by ADP-bound TsaE after the ATP hydrolysis, as TsaE exhibited higher affinity to ADP over ATP (Table 3) [35,102]. In in vitro tRNA t^6^A formation assay, this mechanistic model might work as ATP is supplied in surplus. However, how the inactive ADP-bound TsaE is changed into ATP-bound form to promote the t^6^A-catalysis of TsaD–TsaB in a cellular context remains to be uncovered.

### 4.5. Interaction between tRNA and the KEOPS Complex

The structural models of KEOPS in complex with tRNA demonstrate that all the subunits contribute to the binding of tRNA onto KEOPS (Figure 2C). The models show that ASL of tRNA is lodged into the catalytic center of Kae1/OSGEP with support from Pcc1/LAGE3 and Bud32/TP53RK, which mainly interact with ASL and D-stem of tRNA, respectively. Cgi121/TPRKB could bind to tRNA via accommodating its 3′ CCA end. EMSA assays quantitatively confirmed that *P. abyssi* Pcc1–Kae1 complex forms a binding core for tRNA. The *K_D_* value between tRNA and Pcc1–Kae1 was estimated to be 0.2–0.4 µM, which is comparable to that of the whole KEOPS complex [71]. Similar filter binding experiments with *M. jannaschii* KEOPS proteins showed a strong binding between Bud32–Cgi121 and tRNA, whose binding affinity is comparable to that of either the ternary subcomplex of Kae1–Bud32–Cgi121 or the KEOPS complex [72]. The crystal structure of *M. jannaschii* Cgi121–tRNA reveals that both the 3′ CCA end of tRNA and Cgi121 play critical roles in forming a catalytic tRNA–KEOPS complex [72]. The binding surface and 3′ CCA-coordinating residues (Figure 2B) are conserved in *S. cerevisiae* Cgi121 and *H. sapiens* TPRKB [41,72,100,101]. However, Cgi121 is dispensable in tRNA t^6^A biosynthesis by *P. abyssi* KEOPS [71] or *S. cerevisiae* KEOPS [70]. In particular, Drosophila KEOPS does not even contain a Cgi121 subunit [50]. In addition, *S. cerevisiae* KEOPS catalyzes the formation of t^6^A using tRNAs devoid of 3′ CCA end [27]. These data suggest that an initial recruitment of tRNA by Cgi121 is not strictly required for KEOPS.

Interestingly, an ATP hydrolysis by Bud32 is indispensably involved in t^6^A biosynthesis by yeast and archaean KEOPS [70,71,80,100]. Mutation of the strictly conserved aspartate acid residue (D127 of *P. abyssi* Bud32, D112 of *M. jannaschii* Bud32 and D161 of *S. cerevisiae* Bud32) abolishes ATPase activity of Bud32 and tRNA t^6^A-catalytic activity of KEOPS [70,71,72]. However, the transfer of the TC-moiety from TC-AMP onto A37 of tRNA does not require ATP hydrolysis in terms of chemistry. It turns out that, in an analogy to TsaE in the bacterial TsaD–TsaB–TsaE system, Bud32/TP53RK functions as a “molecular switch” in activating the t^6^A-catalytic activity of KEOPS. Cooperatively, the ATPase activity of Bud32 in KEOPS is strongly potentiated by binding of full-length tRNA but not tRNA devoid of 3′ CCA end [71,72]. NTPases, including ATPases and GTPases, often undergo conformational transitions between the NTP and NDP bound states, of which the slight movement is driven by the energy-releasing hydrolysis of NTP into NDP and Pi [103,104]. In KEOPS machineries, ATP hydrolysis could exert a driving force on the conformational dynamics of the two lobes of Bud32/TP53RK, which may influence its interactions with the substrate tRNAs. Crystal structures and structural models of KEOPS reveal that the C-terminal α-helix of Bud32/TP53RK near the active site of Kae1/OSGEP provides an opportunity to couple the ATP hydrolysis by Bud32/TP53RK and tRNA binding by the KEOPS complex (Figure 2B,C). Based on activity tests, it is therefore hypothesized that the ATP hydrolysis by Bud32/TP53RK drives the dissociation of bound tRNA from KEOPS via reducing its direct interaction between tRNA and itself, or/and promotes the release of t^6^A-tRNA from catalytic centers of Kae1/OSGEP via dislodging the C-terminal tail of Bud32/TP53RK (Figure 2C and Figure 3B) [72,116].

## 5. Diseases Implications

Posttranscriptional modifications of tRNAs regulate the translational efficiency and protein homeostasis [13,52,53,54,55,119]. The frequency of tRNA t^6^A modification varies among different types of cells and is higher in tissues and organs that are less tolerated to the translational deficiency [29,42,46,49,55,56,58,74,80,120,121,122]. In general, tRNA t^6^A and its biosynthetic enzymes (YRDC, KEOPS and OSGEPL1) are more abundant in energy-demanding mitochondria and highly proliferating cells in brain, kidney and liver (Figure 3A) [29,42,56,58,110,114,115]. The depletion of tRNA t^6^A leads to cell death or severe growing phenotypes via causing glitches of translation apparatus in the cytosol and in mitochondria [26,59,60,110]. In clinical cases, deregulation of tRNA t^6^A-biosynthetic machineries has been implicated in patients with mitochondrial diseases [29] and individuals with a range of congenital nephrotic syndromes [121,122], including GAMOS (Table 5) [42,80,111,123,124].

### 5.1. Mitochondrial Diseases Caused by tRNA t^6^A

In human mitochondria, tRNA t^6^A functions to monitor hypocarbia and regulate a set of genes involved in the Warburg effect, which occurs in order to better metabolize glucose anaerobically in highly proliferating cancer cells [13,29]. Knockout or knockdown of OSGEPL1 and YRDC deprives five mitochondrial tRNAs (Ile, Lys, Asn, Ser and Thr) of t^6^A modification, leading to respiratory defects in mitochondria [29,125]. Specifically, the t^6^A modification frequency of mitochondrial tRNAs is down-regulated by low cellular concentration of CO_2_/HCO_3_^−^ in conditions of hypoxia [29]. The pathogenic mutations, A37G in mitochondrial tRNA^Lys^ and tRNA^Asn^, abolished t^6^A formation, caused codon-specific dysfunction in mitochondrial translation, resulting in mitochondrial cytopathy and mitochondrial encephalopathy, respectively [29,30,53]. Mitochondrial tRNA^Thr^ isolated from a myoclonic epilepsy with ragged red fibers (MERRF) patient contains significantly low levels of t^6^A modification [29,55]. The mitochondrial tRNA^Thr^ from individuals with MERRF harbors pathogenic mutations on the U36A37A38 motif, which was mutated into U36A37G38 and resulted in deficiency of t^6^A modification [29]. By contrast, mitochondrial tRNA^Ile^ from individuals with Leigh syndrome contains so-called “gain-of-function” mutations-U36A37G38 to U36A37A38, which led to unwanted t^6^A-modification of tRNA^Ile^ [29]. In addition, the mutation of CDKAL1 gene causes type 2 diabetes, for reasons that CDKAL1 dysfunction results in aberration of the ms^2^t^6^A modification of cytoplasmic tRNA^Lys^, which leads to misreading of lysine codons in proinsulin [54,57,58,119].

### 5.2. KEOPS Mutations and Neurological Disorders

KOEPS is universally expressed in the human body and plays an essential role in maintaining the fitness of humans [114,115,126,127]. In particular, KOEPS-encoding genes are highly expressed in pronephros and in neural tissue such as the developing brain, eye and cranial cartilage (Figure 3A) [127]. In human podocytes, the knockdown of KEOPS subunit genes led to the loss of tRNA t^6^A, interfered with protein biosynthesis and led to activation of the unfolded protein response and endoplasmic reticulum stress [56]. In clinical studies, recessive pathogenic mutations in KEOPS-encoding genes have been found in sequence genomes of patients with GAMOS (Figure 3A and Table 5) [42,56,80,111,121,124]. Individuals with GAMOS also contain pathogenic missense variants of YRDC [42] and WDR4-a tRNA 7-methylguanosine (m^7^G) synthetase [128,129]. These genotype-phenotype analyses suggested that the loss of tRNA t^6^A modification, and probably other essential tRNA modifications, is implicated in the pathogenesis of GAMOS via causing deregulation of the translation processes. Except for a number of nonsense mutations leading to translation termination, most GAMOS related missense mutations are projected onto the crystal structures of GON7–LAGE3–OSGEP [42] and TP53RK–TPRKB [100], and the structural model of the human KEOPS-tRNA complex. The exact positions and possible effects of mutations on the structure and function of KEOPS are summarized in Table 5 and Figure 3B. While a number of mutations in OSGEP and TP53RK may interfere with the t^6^A-catalytic activity of OSGEP and ATPase activity of TP53RK, others may affect the assembly of a catalytically active KEOPS and possibly an expanded protein interaction network involving KEOPS [56,79].

## 6. Conclusions and Future Perspectives

As one of the most chemically and biosynthetically complex modifications, tRNA t^6^A plays an essential role in promoting translational efficiency and in regulating protein homeostasis. Depletion of tRNA t^6^A leads to pleiotropic phenotypes in single cell organisms and a variety of pathological consequences in higher eukaryotes. The biosynthesis of tRNA t^6^A is catalyzed by two ancient protein families of TsaC/Sua5 and TsaD/Kae1/Qri7, which belong to LUCA. Except for the prototypal mitochondrial system made up of TsaC/Sua5 and Qri7/OSGEPL1 proteins, TsaD/Kae1/OSGEP proteins require accessory proteins to catalyze the biosynthesis of tRNA t^6^A. While a conservation in the chemical transformation and catalytic pathway has been sustained in both TsaC/Sua5 protein and TsaD/Kae1/Qri7 proteins, the mechanisms in catalytic activation of TsaD/Kae1/Qri7 proteins have been diversified over evolution. It is hypothesized that higher orders of assembly enable more precise regulations of the catalytic machineries, which are definitely demanded to cope with the increasing complexity of biological systems. In particular, the ATP hydrolysis-based regulation of the TsaD–TsaB–TsaE and KEOPS complex allows more efficient catalysis and well-tuned regulations in a spatiotemporal fashion. However, a number of fundamental questions pertaining to the t^6^A catalysis, including the recognition of A37 and the catalytic events, await elucidation.

The evolutionary relationship and cellular roles of the KEOPS complex are still rather enigmatic. The composition of KEOPS in archaea and eukaryotes differs mainly in Gon7/GON7, which are only found in yeast and humans. Yeast Gon7 and human GON7 are intrinsically disordered proteins. They are not evolutionarily related but function as structural analogs in the KEOPS assembly. In addition, Drosophila KEOPS only consists of Kae1, Bud32 and Pcc1. The absence of Cgi121 in Drosophila defies the molecular working model in which Cgi121 plays an essential role in recruiting tRNA onto KEOPS. Furthermore, it appears that KEOPS or its subunits may perform other functions independent of that in catalyzing the tRNA t^6^A modification. Unlike yeast KEOPS, which is imported into the nucleus and promotes telomere uncapping and elongation independent of tRNA t^6^A modification, human KEOPS is localized in the cytoplasm and is not functionally involved in telomere replication. Future work will be needed in investigating the seemingly diverse cellular roles of KEOPS and in dissecting the molecular pathways in mammalian development.

Protein synthesis is the connecting knot that links the molecular roles tRNA t^6^A to cellular life and pathological consequences. Deregulation in protein synthesis in both the cytosol and mitochondria can cause a plethora of disastrous consequences, which are also dependent on the codon usage and the tolerance of tRNA t^6^A depletion. However, it is hard to dissect the molecular layers at which the dysfunction in tRNA t^6^A biosynthetic machineries is causing problems and various pleiotropic phenotypes in bacteria, yeast and higher eukaryotes. Pathogenic mutations of human YRDC and KEOPS are implicated in the pathogenesis of the autosomal recessive GAMOS via interfering with the protein synthesis in the absence of tRNA t^6^A. Nonetheless, a dysfunction in protein synthesis could be attributed to the loss of other tRNA modifications (i.e., m^7^G) in patients with GAMOS. In addition, depletion and hypomodification of tRNA t^6^A have occurred in individuals with mitochondrial diseases such as MERRF. However, it is still challenging to convince medical doctors of the validity or feasibility of KEOPS as a target for medical interference of neurological disorders, which may involve mutations of other genes. Therefore, causative roles of YRDC, OSGEPL1 and KEOPS in specific types of diseases or physiological disorders await in-depth analyses. A t^6^A-based quantitative sequencing technique is desirable, analyzing the frequency of tRAN t^6^A under physiological and pathological conditions.

Last but not least, one would have marveled at the achievement of elucidating the tRNA t^6^A biosynthesis over the past five decades. In retrospect, a great number of talented colleagues have contributed to experimental data aggregation and have offered insightful ideas toward resolving the enzymatic pathways and molecular workings of tRNA t^6^A modification in the three domains of life. In particular, several breakthroughs have played key roles in advancing this line of research: (i) establishment of a biosynthetic relationship between the universally distributed but function-unknown LUCA families (TsaC/Sua5 and TsaD/Kae1) and the universally found tRNA t^6^A modification without known enzymes; (ii) discovery and structural analyses of KEOPS/EKC; (iii) biochemical and biophysical analyses on TsaD–TsaB–TsaE interaction network; (iv) clinical genotype and phenotype analyses on mutations of YRDC and KEOPS in human diseases. In the future, we expect to see more breakthroughs in the mechanistic understanding of the roles of tRNA t^6^A and its biosynthetic enzymes.

## Figures and Tables

**Table 2:** Summary of the binding between the t^6^A-modifying enzymes and ligands. Abbreviations: *Ec*, *Escherichia coli*; *St*, *Sulfolobus tokodaii*; *Tm*, *Thermotoga maritima*; *Hi*, *Haemophilus influenzae*; *Hs*, *H. sapiens*; FQ, fluorescence quenching; ITC, isothermal titration calorimetry; MST, microscale thermophoresis; FRET, fluorescence resonance energy transfer; TsaBD, TsaD–TsaB; TsaDBE, TsaD–TsaB–TsaE; *, *K_M_* values.

Enzymes	Ligands	*K_D_* (μM)	Methods	Refs
*Ec*TsaC	ATP	1.04 ± 0.67	FQ	[83]
	*L*-Threonine	0.04 ± 0.10		
*St*Sua5	ATP	61.3 ± 3.0	ITC	[33]
	ADP	101.0 ± 3.3		
	AMP	1420 ± 170		
	*L*-Threonine	9.3 ± 0.3		
*Hs*YRDC	ATP	170 ± 220 *	Kinetic analysis	[29]
	*L*-Threonine	190 ± 60 *		
	HCO_3_^−^	13000 ± 3800 *		
*Ec*TsaD	AMPCPP	1.62 ± 0.088	ITC	[35]
*Ec*TsaDB	AMPCPP	0.70 ± 0.011	ITC	[35]
	BK951	3.4 ± 0.6	MST	[36]
*Ec*TsaE	MANT-ADP	8 ± 0.9	FRET	[84]
	ADP	6.54 ± 0.053	ITC	[35]
	ATPγS	20.41 ± 1.32	ITC	[35]
*Tm*TsaDBE	AMPPCP	6.1 ± 1.0	ITC	[85]
*Tm*TsaE	AMPPCP	9.0 ± 1.3	ITC	[85]
*Hi*TsaE	MANT-ADP	18 ± 0.4	FRET	[84]

**Table 3:** Summary of enzymatically validated tRNA t^6^A-modifying enzymes and tRNA substrates. Abbreviations: *Bs*, *Bacillus subtilis*; *Sc*, *Saccharomyces cerevisiae*; *Hs*, *Homo sapiens*; *Ec*, *Escherichia coli*; *Pa*, *Pyrococcus abyssi*; *Mj*, *Methanocaldococcus jannaschii*; *Tm*, *Thermotoga maritima*; *Ce*, *Caenorhabditis elegans*; mt, mitochondrial; TsaBDE, TsaD–TsaB–TsaE.

Organisms	Enzymes [Refs]	t^6^A-Modified tRNAs
Bacteria	*Bs*TsaC and *Bs*TsaBDE [79]	*Bs*tRNA^Lys^ (UUU) and *Bs*tRNA^Thr^ (GGU)
	*Sc*Sua5/*Pa*Sua5/*Ec*TsaC and *Ec*TsaBDE [17,18,27,29,66,72]	*Hs*mt-tRNA^Asn^ (GUU), *Hs*mt-tRNA^Thr^ (UGU), *Hs*mt-tRNA^Ile^ (GAU), *Hs*mt-tRNA^Ser (AGY)^(GCU), *Hs*mt-tRNA^Lys^ (UUU) *Ec*tRNA^iMet^ (CAU), *Ec*tRNA^Ile^ (GAU), *Ec*tRNA^Lys^ (UUU), *Ec*tRNA^Asn^ (GUU), *Mj*tRNA^Lys^ (UUU) and *Ec*tRNA^Thr^ (GGU)
	*Tm*TsaC and *Tm*TsaBDE [38,85]	*Ec*tRNA^Thr^ (CGU) and *Ec*tRNA^Lys^ (UUU)
Archaea	*Sc*Sua5/*Pa*Sua5/*Ec*TsaC and *Pa*KEOPS [65]	*Ec*tRNA^Asn^ (GUU), *Ec*tRNA^Ile^ (GAU) *Ec*tRNA^fMet^ (CAU), *Ec*tRNA^Lys^ (UUU), *Pa*tRNA^Lys^ (UUU) and *Pa*tRNA^Ile^ (GAU)
	*Mj*Sua5 and *Mj*KEOPS [72]	*Mj*tRNA^Asn^ (GUU), *Mj*tRNA^Met^ (CAU), *Sc*tRNA^Ile^ (AAU), *Mj*tRNA^Lys^ (UUU) and *Mj*tRNA^Thr^ (GGU)
Eukarya	*Sc*Sua5/*Pa*Sua5/*Ec*TsaC/*Bs*TsaC and *Sc*KEOPS [18,27,65,74]	*Hs*tRNA^Asn^ (GUU), *Hs*tRNA^Ser^ (GCU), *Hs*tRNA^Lys^ (CUU/UUU), *Hs*tRNA^iMet^ (CAU), *Hs*tRNA^Arg^ (CCU/UCU), *Hs*tRNA^Thr^ (AGU/CGU/UGU), *Sc*tRNA^iMet^ (CAU), *Sc*mt-tRNA^Arg^ (UCU), *Hs*tRNA^Ile^ (AAU/GAU/UAU), *Ec*tRNA^Lys^ (UUU), *Ec*tRNA^Asn^ (GUU), *Ec*tRNA^Thr^ (AGU/CGU/UGU) and *Hs*mt-tRNA^Thr^ (UGU)
	*Hs*YRDC and *Ce*KEOPS [27]	*Hs*tRNA^iMet^ (CAU) and *Hs*tRNA^Thr^ (UGU)
	*Hs*YRDC and *Hs*KEOPS [27,72,79]	*Hs*tRNA^Ile^ (UAU), *Hs*tRNA^iMet^ (CAU), *Hs*tRNA^Thr^ (UGU), *Hs*tRNA^Arg^ (UCU), *Hs*tRNA^Lys^ (UUU) and *Sc*mt-tRNA^Arg^ (UCU)
	*Sc*Sua5/*Ec*TsaC and *Sc*Qri7 [18,72,74]	*Ec*tRNA^Lys^ (UUU), *Mj*tRNA^Lys^ (UUU), *Sc*tRNA^Ile^ (AAU), *Hs*mt-tRNA^Thr^ (UGU) and *Sc*mt-tRNA^Arg^ (UCU)
	*Hs*YRDC and *Hs*OSGEPL1 [29,74]	*Hs*mt-tRNA^Ser (AGY)^(GCU), *Hs*mt-tRNA^Lys^ (UUU), *Hs*mt-tRNA^Ile^ (GAU), *Hs*mt-tRNA^Asn^ (GUU) and *Hs*mt-tRNA^Thr^ (UGU)

**Table 4:** Summary of *K_D_* values of the binding between tRNA t^6^A-modifying enzymes and tRNAs. Abbreviations: *Ec*, *E. coli*; *Tm*, *T. maritima*; *Pa*, *P. abyssi*; *Mj*, *M. jannaschii*; *Sc*, *S. cerevisiae*; *Hs*, *H. sapiens*; mt, mitochondrial; FQ, fluorescence quenching; EMSA, electrophoretic mobility shift assay; FP, fluorescence polarization; BLI, biolayer interferometry; TsaDB, TsaD–TsaB; TsaB_2_D_2_, TsaD–TsaB-TsaB–TsaD; TsaB_2_D_2_E, TsaD–TsaB–TsaB–TsaD–TsaE; KEOPS, Cgi121–Bud32–Kae1–Pcc1; KBC, Kae1–Bud32–Cgi121; BC, Bud32–Cgi121; * total tRNAs purified from *E. coli*.

Proteins	tRNAs	*K_D_* (μM)	Methods	Refs
*Ec*TsaC	Modified *Ec*tRNA^Thr^ (CGU)	0.62 ± 0.13	FQ	[59]
	*Ec*tRNA^Thr^ (CGU)	0.11 ± 0.05		[59]
	*Ec*tRNAs *	0.68 ± 0.15		[31]
	*Ec*ASL^Lys^ (UUU)	0.27 ± 0.20		[83]
*Ec*TsaDB	*Ec*tRNA^Lys^ (UUU)	0.087 ± 0.011	FQ	[36]
*Tm*TsaB_2_D_2_	*Ec*tRNA^Thr^ (CGU)	1.3 ± 0.07	EMSA	[85]
*Tm*TsaB_2_D_2_E	*Ec*tRNA^Thr^ (CGU)	0.8 ± 0.02		
*Pa*KEOPS	*Ec*tRNA^Lys^ (UUU)	0.1~0.5	EMSA	[65]
*Pa*Pcc1–Kae1		0.2~0.4		
*Pa*Kae1–Bud32		~2.0		
*Pa*Cgi121		No binding		
*Mj*KEOPS	*Mj*tRNA^Lys^ (UUU)	0.263 ± 0.064	FP	[72]
*Mj*KBC		0.153 ± 0.068		
*Mj*BC		0.229 ± 0.027		
*Mj*Cgi121		0.730 ± 0.060		
*Hs*KEOPS	*Mj*tRNA^Lys^ (UUU)	4.30 ± 0.64	FP	[72]
*Hs*BC		0.45 ± 0.05		
*Hs*TPRKB		28.3 ± 10		
*Sc*Qri7–Qri7	*Hs*mt-tRNA^Thr^ (CGU)	0.0269 ± 0.0012	BLI	[30]
*Hs*OSGEPL1	*Hs*mt-tRNA^Thr^ (UGU)	6.7 ± 0.1	BLI	[74]

**Table 5:** KEOPS-related mutations identified in individuals with GAMOS.

Proteins	Mutations	Effect	Refs
OSGEP	Ile14Phe Cys110Arg Ile111Thr	May affect catalytic activity of OSGEP	[56,124]
	Lys78Glu Val107Met	May interfere with protein folding	[56]
	Lys198Arg Arg247Gln Arg280His	May interfere with the interaction with TP53RK	[42,124]
	Arg325Gln	May interfere with the interaction with the tRNA substrate	[56]
TP53RK	Gly42Asp	May affect the ATPase activity of TP53RK	[80]
	Arg55Gly Lys60Ser Thr81Arg	May interfere with the interaction with TPRKB	[56,80,100]
	Lys65Met	May interfere with the interaction with OSGEP	[100,111]
	Arg243Leu	May affect the t^6^A-catalytic activity of OSGEP	[100]
TPRKB	Leu136Pro Tyr149Cys	May affect protein structural integrity	[100]
LAGE3	Val106Phe	May affect protein structural integrity	[56]
	Phe137Ser	May interfere with the interaction with OSGEP	[42]
GON7	Try7*	Loss of GON7, affects KEOPS stability	[42]

## References

[1] Crick F. (1970). Central dogma of molecular biology. Nature.

[2] Moore P.B., Steitz T.A. (2011). The roles of RNA in the synthesis of protein. Cold Spring Harb. Perspect. Biol..

[3] Jackman J.E., Alfonzo J.D. (2013). Transfer RNA modifications: Nature’s combinatorial chemistry playground. Wiley Interdiscip. Rev. RNA.

[4] Boccaletto P., Stefaniak F., Ray A., Cappannini A., Mukherjee S., Purta E., Kurkowska M., Shirvanizadeh N., Destefanis E., Groza P. (2022). MODOMICS: A database of RNA modification pathways. 2021 update. Nucleic Acids Res..

[5] Durant P.C., Bajji A.C., Sundaram M., Kumar R.K., Davis D.R. (2005). Structural effects of hypermodified nucleosides in the *Escherichia coli* and human tRNALys anticodon loop: The effect of nucleosides s2U, mcm5U, mcm5s2U, mnm5s2U, t6A, and ms2t6A. Biochemistry.

[6] Motorin Y., Helm M. (2010). tRNA stabilization by modified nucleotides. Biochemistry.

[7] Yarian C., Townsend H., Czestkowski W., Sochacka E., Malkiewicz A.J., Guenther R., Miskiewicz A., Agris P.F. (2002). Accurate translation of the genetic code depends on tRNA modified nucleosides. J. Biol. Chem..

[8] Alexandrov A., Chernyakov I., Gu W., Hiley S.L., Hughes T.R., Grayhack E.J., Phizicky E.M. (2006). Rapid tRNA decay can result from lack of nonessential modifications. Mol. Cell.

[9] Helm M. (2006). Post-transcriptional nucleotide modification and alternative folding of RNA. Nucleic Acids Res..

[10] Phizicky E.M., Hopper A.K. (2010). tRNA biology charges to the front. Genes Dev..

[11] Grosjean H., Sprinzl M., Steinberg S. (1995). Posttranscriptionally modified nucleosides in transfer RNA: Their locations and frequencies. Biochimie.

[12] El Yacoubi B., Bailly M., de Crecy-Lagard V. (2012). Biosynthesis and function of posttranscriptional modifications of transfer RNAs. Annu. Rev. Genet..

[13] Suzuki T. (2021). The expanding world of tRNA modifications and their disease relevance. Nat. Rev. Mol. Cell Biol..

[14] Agris P.F., Vendeix F.A., Graham W.D. (2007). tRNA’s wobble decoding of the genome: 40 years of modification. J. Mol. Biol..

[15] Chheda G.B. (1969). Isolation and characterization of a novel nucleoside, N-[(9-beta-D-ribofuranosyl-9H-purin-6-yl)carbamoyl]threonine, from human urine. Life Sci..

[16] Schweizer M.P., Chheda G.B., Baczynskyj L., Hall R.H. (1969). Aminoacyl nucleosides. VII. N-(Purin-6-ylcarbamoyl)threonine. A new component of transfer ribonucleic acid. Biochemistry.

[17] Thiaville P.C., El Yacoubi B., Kohrer C., Thiaville J.J., Deutsch C., Iwata-Reuyl D., Bacusmo J.M., Armengaud J., Bessho Y., Wetzel C. (2015). Essentiality of threonylcarbamoyladenosine (t(6)A), a universal tRNA modification, in bacteria. Mol. Microbiol..

[18] Thiaville P.C., El Yacoubi B., Perrochia L., Hecker A., Prigent M., Thiaville J.J., Forterre P., Namy O., Basta T., de Crecy-Lagard V. (2014). Cross kingdom functional conservation of the core universally conserved threonylcarbamoyladenosine tRNA synthesis enzymes. Eukaryot. Cell.

[19] Parthasarathy R., Ohrt J.M., Chheda G.B. (1977). Modified nucleosides and conformation of anticodon loops: Crystal structure of t6A and g6A. Biochemistry.

[20] Sundaram M., Durant P.C., Davis D.R. (2000). Hypermodified nucleosides in the anticodon of tRNALys stabilize a canonical U-turn structure. Biochemistry.

[21] Han L., Phizicky E.M. (2018). A rationale for tRNA modification circuits in the anticodon loop. Rna.

[22] Murphy F.V.t., Ramakrishnan V., Malkiewicz A., Agris P.F. (2004). The role of modifications in codon discrimination by tRNA(Lys)UUU. Nat. Struct. Mol. Biol..

[23] Weissenbach J., Grosjean H. (1981). Effect of threonylcarbamoyl modification (t6A) in yeast tRNA Arg III on codon-anticodon and anticodon-anticodon interactions. A thermodynamic and kinetic evaluation. Eur. J. Biochem..

[24] Konevega A.L., Soboleva N.G., Makhno V.I., Semenkov Y.P., Wintermeyer W., Rodnina M.V., Katunin V.I. (2004). Purine bases at position 37 of tRNA stabilize codon-anticodon interaction in the ribosomal A site by stacking and Mg2+-dependent interactions. Rna.

[25] Lescrinier E., Nauwelaerts K., Zanier K., Poesen K., Sattler M., Herdewijn P. (2006). The naturally occurring N6-threonyl adenine in anticodon loop of Schizosaccharomyces pombe tRNAi causes formation of a unique U-turn motif. Nucleic Acids Res..

[26] Thiaville P.C., Legendre R., Rojas-Benitez D., Baudin-Baillieu A., Hatin I., Chalancon G., Glavic A., Namy O., de Crecy-Lagard V. (2016). Global translational impacts of the loss of the tRNA modification t(6)A in yeast. Microb. Cell.

[27] Wang J.T., Zhou J.B., Mao X.L., Zhou L., Chen M., Zhang W., Wang E.D., Zhou X.L. (2022). Commonality and diversity in tRNA substrate recognition in t6A biogenesis by eukaryotic KEOPSs. Nucleic Acids Res..

[28] Nagao A., Ohara M., Miyauchi K., Yokobori S.I., Yamagishi A., Watanabe K., Suzuki T. (2017). Hydroxylation of a conserved tRNA modification establishes non-universal genetic code in echinoderm mitochondria. Nat. Struct. Mol. Biol..

[29] Lin H., Miyauchi K., Harada T., Okita R., Takeshita E., Komaki H., Fujioka K., Yagasaki H., Goto Y.I., Yanaka K. (2018). CO2-sensitive tRNA modification associated with human mitochondrial disease. Nat. Commun..

[30] Wang Y., Zeng Q.Y., Zheng W.Q., Ji Q.Q., Zhou X.L., Wang E.D. (2018). A natural non-Watson-Crick base pair in human mitochondrial tRNAThr causes structural and functional susceptibility to local mutations. Nucleic Acids Res..

[31] Teplova M., Tereshko V., Sanishvili R., Joachimiak A., Bushueva T., Anderson W.F., Egli M. (2000). The structure of the yrdC gene product from *Escherichia coli* reveals a new fold and suggests a role in RNA binding. Protein Sci..

[32] Pichard-Kostuch A., Zhang W., Liger D., Daugeron M.C., Letoquart J., Li de la Sierra-Gallay I., Forterre P., Collinet B., van Tilbeurgh H., Basta T. (2018). Structure-function analysis of Sua5 protein reveals novel functional motifs required for the biosynthesis of the universal t(6)A tRNA modification. RNA.

[33] Kuratani M., Kasai T., Akasaka R., Higashijima K., Terada T., Kigawa T., Shinkai A., Bessho Y., Yokoyama S. (2011). Crystal structure of Sulfolobus tokodaii Sua5 complexed with L-threonine and AMPPNP. Proteins.

[34] Parthier C., Gorlich S., Jaenecke F., Breithaupt C., Brauer U., Fandrich U., Clausnitzer D., Wehmeier U.F., Bottcher C., Scheel D. (2012). The O-carbamoyltransferase TobZ catalyzes an ancient enzymatic reaction. Angew. Chem. Int. Ed..

[35] Zhang W., Collinet B., Perrochia L., Durand D., van Tilbeurgh H. (2015). The ATP-mediated formation of the YgjD-YeaZ-YjeE complex is required for the biosynthesis of tRNA t6A in *Escherichia coli*. Nucleic Acids Res..

[36] Kopina B.J., Missoury S., Collinet B., Fulton M.G., Cirio C., van Tilbeurgh H., Lauhon C.T. (2021). Structure of a reaction intermediate mimic in t6A biosynthesis bound in the active site of the TsaBD heterodimer from *Escherichia coli*. Nucleic Acids Res..

[37] Nichols C.E., Lamb H.K., Thompson P., El Omari K., Lockyer M., Charles I., Hawkins A.R., Stammers D.K. (2013). Crystal structure of the dimer of two essential Salmonella typhimurium proteins, YgjD & YeaZ and calorimetric evidence for the formation of a ternary YgjD-YeaZ-YjeE complex. Protein Sci..

[38] Luthra A., Paranagama N., Swinehart W., Bayooz S., Phan P., Quach V., Schiffer J.M., Stec B., Iwata-Reuyl D., Swairjo M.A. (2019). Conformational communication mediates the reset step in t6A biosynthesis. Nucleic Acids Res..

[39] Wan L.C., Pillon M.C., Thevakumaran N., Sun Y., Chakrabartty A., Guarne A., Kurinov I., Durocher D., Sicheri F. (2016). Structural and functional characterization of KEOPS dimerization by Pcc1 and its role in t6A biosynthesis. Nucleic Acids Res..

[40] Hecker A., Leulliot N., Gadelle D., Graille M., Justome A., Dorlet P., Brochier C., Quevillon-Cheruel S., Le Cam E., van Tilbeurgh H. (2007). An archaeal orthologue of the universal protein Kae1 is an iron metalloprotein which exhibits atypical DNA-binding properties and apurinic-endonuclease activity in vitro. Nucleic Acids Res..

[41] Mao D.Y., Neculai D., Downey M., Orlicky S., Haffani Y.Z., Ceccarelli D.F., Ho J.S., Szilard R.K., Zhang W., Ho C.S. (2008). Atomic structure of the KEOPS complex: An ancient protein kinase-containing molecular machine. Mol. Cell.

[42] Arrondel C., Missoury S., Snoek R., Patat J., Menara G., Collinet B., Liger D., Durand D., Gribouval O., Boyer O. (2019). Defects in t(6)A tRNA modification due to GON7 and YRDC mutations lead to Galloway-Mowat syndrome. Nat. Commun..

[43] Tominaga T., Kobayashi K., Ishii R., Ishitani R., Nureki O. (2014). Structure of *Saccharomyces cerevisiae* mitochondrial Qri7 in complex with AMP. Acta Crystallogr. Sect. F Struct. Biol. Commun..

[44] Petkun S., Shi R., Li Y., Asinas A., Munger C., Zhang L., Waclawek M., Soboh B., Sawers R.G., Cygler M. (2011). Structure of hydrogenase maturation protein HypF with reaction intermediates shows two active sites. Structure.

[45] Matuszewski M., Wojciechowski J., Miyauchi K., Gdaniec Z., Wolf W.M., Suzuki T., Sochacka E. (2017). A hydantoin isoform of cyclic N6-threonylcarbamoyladenosine (ct6A) is present in tRNAs. Nucleic Acids Res..

[46] Miyauchi K., Kimura S., Suzuki T. (2013). A cyclic form of N6-threonylcarbamoyladenosine as a widely distributed tRNA hypermodification. Nat. Chem. Biol..

[47] Arragain S., Handelman S.K., Forouhar F., Wei F.Y., Tomizawa K., Hunt J.F., Douki T., Fontecave M., Mulliez E., Atta M. (2010). Identification of eukaryotic and prokaryotic methylthiotransferase for biosynthesis of 2-methylthio-N6-threonylcarbamoyladenosine in tRNA. J. Biol. Chem..

[48] Kimura S., Miyauchi K., Ikeuchi Y., Thiaville P.C., Crecy-Lagard V., Suzuki T. (2014). Discovery of the beta-barrel-type RNA methyltransferase responsible for N6-methylation of N6-threonylcarbamoyladenosine in tRNAs. Nucleic Acids Res..

[49] Kang B.I., Miyauchi K., Matuszewski M., D’Almeida G.S., Rubio M.A.T., Alfonzo J.D., Inoue K., Sakaguchi Y., Suzuki T., Sochacka E. (2017). Identification of 2-methylthio cyclic N6-threonylcarbamoyladenosine (ms2ct6A) as a novel RNA modification at position 37 of tRNAs. Nucleic Acids Res..

[50] Rojas-Benitez D., Ibar C., Glavic A. (2013). The Drosophila EKC/KEOPS complex: Roles in protein synthesis homeostasis and animal growth. Fly.

[51] Lin C.J., Smibert P., Zhao X., Hu J.F., Ramroop J., Kellner S.M., Benton M.A., Govind S., Dedon P.C., Sternglanz R. (2015). An extensive allelic series of Drosophila kae1 mutants reveals diverse and tissue-specific requirements for t6A biogenesis. RNA.

[52] Chujo T., Tomizawa K. (2021). Human transfer RNA modopathies: Diseases caused by aberrations in transfer RNA modifications. FEBS J..

[53] Tahmasebi S., Khoutorsky A., Mathews M.B., Sonenberg N. (2018). Translation deregulation in human disease. Nat. Rev. Mol. Cell Biol..

[54] Pereira M., Francisco S., Varanda A.S., Santos M., Santos M.A.S., Soares A.R. (2018). Impact of tRNA Modifications and tRNA-Modifying Enzymes on Proteostasis and Human Disease. Int. J. Mol. Sci..

[55] Suzuki T., Nagao A., Suzuki T. (2011). Human mitochondrial tRNAs: Biogenesis, function, structural aspects, and diseases. Annu. Rev. Genet..

[56] Braun D.A., Rao J., Mollet G., Schapiro D., Daugeron M.C., Tan W., Gribouval O., Boyer O., Revy P., Jobst-Schwan T. (2017). Mutations in KEOPS-complex genes cause nephrotic syndrome with primary microcephaly. Nat. Genet..

[57] Steinthorsdottir V., Thorleifsson G., Reynisdottir I., Benediktsson R., Jonsdottir T., Walters G.B., Styrkarsdottir U., Gretarsdottir S., Emilsson V., Ghosh S. (2007). A variant in CDKAL1 influences insulin response and risk of type 2 diabetes. Nat. Genet..

[58] Wei F.Y., Suzuki T., Watanabe S., Kimura S., Kaitsuka T., Fujimura A., Matsui H., Atta M., Michiue H., Fontecave M. (2011). Deficit of tRNA(Lys) modification by Cdkal1 causes the development of type 2 diabetes in mice. J. Clin. Investig..

[59] El Yacoubi B., Lyons B., Cruz Y., Reddy R., Nordin B., Agnelli F., Williamson J.R., Schimmel P., Swairjo M.A., de Crecy-Lagard V. (2009). The universal YrdC/Sua5 family is required for the formation of threonylcarbamoyladenosine in tRNA. Nucleic Acids Res..

[60] El Yacoubi B., Hatin I., Deutsch C., Kahveci T., Rousset J.P., Iwata-Reuyl D., Murzin A.G., de Crecy-Lagard V. (2011). A role for the universal Kae1/Qri7/YgjD (COG0533) family in tRNA modification. EMBO J..

[61] Koonin E.V. (2003). Comparative genomics, minimal gene-sets and the last universal common ancestor. Nat. Rev. Microbiol..

[62] Arigoni F., Talabot F., Peitsch M., Edgerton M.D., Meldrum E., Allet E., Fish R., Jamotte T., Curchod M.L., Loferer H. (1998). A genome-based approach for the identification of essential bacterial genes. Nat. Biotechnol..

[63] Butland G., Peregrin-Alvarez J.M., Li J., Yang W., Yang X., Canadien V., Starostine A., Richards D., Beattie B., Krogan N. (2005). Interaction network containing conserved and essential protein complexes in *Escherichia coli*. Nature.

[64] Galperin M.Y., Koonin E.V. (2004). ‘Conserved hypothetical’ proteins: Prioritization of targets for experimental study. Nucleic Acids Res..

[65] Perrochia L., Crozat E., Hecker A., Zhang W., Bareille J., Collinet B., van Tilbeurgh H., Forterre P., Basta T. (2013). In vitro biosynthesis of a universal t6A tRNA modification in Archaea and Eukarya. Nucleic Acids Res..

[66] Deutsch C., El Yacoubi B., de Crecy-Lagard V., Iwata-Reuyl D. (2012). Biosynthesis of threonylcarbamoyl adenosine (t6A), a universal tRNA nucleoside. J. Biol. Chem..

[67] Lauhon C.T. (2012). Mechanism of N6-threonylcarbamoyladenonsine (t(6)A) biosynthesis: Isolation and characterization of the intermediate threonylcarbamoyl-AMP. Biochemistry.

[68] Downey M., Houlsworth R., Maringele L., Rollie A., Brehme M., Galicia S., Guillard S., Partington M., Zubko M.K., Krogan N.J. (2006). A genome-wide screen identifies the evolutionarily conserved KEOPS complex as a telomere regulator. Cell.

[69] Kisseleva-Romanova E., Lopreiato R., Baudin-Baillieu A., Rousselle J.C., Ilan L., Hofmann K., Namane A., Mann C., Libri D. (2006). Yeast homolog of a cancer-testis antigen defines a new transcription complex. EMBO J..

[70] Srinivasan M., Mehta P., Yu Y., Prugar E., Koonin E.V., Karzai A.W., Sternglanz R. (2011). The highly conserved KEOPS/EKC complex is essential for a universal tRNA modification, t6A. EMBO J..

[71] Perrochia L., Guetta D., Hecker A., Forterre P., Basta T. (2013). Functional assignment of KEOPS/EKC complex subunits in the biosynthesis of the universal t6A tRNA modification. Nucleic Acids Res..

[72] Beenstock J., Ona S.M., Porat J., Orlicky S., Wan L.C.K., Ceccarelli D.F., Maisonneuve P., Szilard R.K., Yin Z., Setiaputra D. (2020). A substrate binding model for the KEOPS tRNA modifying complex. Nat. Commun..

[73] Wan L.C., Mao D.Y., Neculai D., Strecker J., Chiovitti D., Kurinov I., Poda G., Thevakumaran N., Yuan F., Szilard R.K. (2013). Reconstitution and characterization of eukaryotic N6-threonylcarbamoylation of tRNA using a minimal enzyme system. Nucleic Acids Res..

[74] Zhou J.B., Wang Y., Zeng Q.Y., Meng S.X., Wang E.D., Zhou X.L. (2020). Molecular basis for t6A modification in human mitochondria. Nucleic Acids Res..

[75] Harris K.A., Bobay B.G., Sarachan K.L., Sims A.F., Bilbille Y., Deutsch C., Iwata-Reuyl D., Agris P.F. (2015). NMR-based Structural Analysis of Threonylcarbamoyl-AMP Synthase and Its Substrate Interactions. J. Biol. Chem..

[76] Agari Y., Sato S., Wakamatsu T., Bessho Y., Ebihara A., Yokoyama S., Kuramitsu S., Shinkai A. (2008). X-ray crystal structure of a hypothetical Sua5 protein from Sulfolobus tokodaii strain 7. Proteins.

[77] Naor A., Thiaville P.C., Altman-Price N., Cohen-Or I., Allers T., de Crecy-Lagard V., Gophna U. (2012). A genetic investigation of the KEOPS complex in halophilic Archaea. PLoS ONE.

[78] Oberto J., Breuil N., Hecker A., Farina F., Brochier-Armanet C., Culetto E., Forterre P. (2009). Qri7/OSGEPL, the mitochondrial version of the universal Kae1/YgjD protein, is essential for mitochondrial genome maintenance. Nucleic Acids Res..

[79] Wan L.C., Maisonneuve P., Szilard R.K., Lambert J.P., Ng T.F., Manczyk N., Huang H., Laister R., Caudy A.A., Gingras A.C. (2017). Proteomic analysis of the human KEOPS complex identifies C14ORF142 as a core subunit homologous to yeast Gon7. Nucleic Acids Res..

[80] Treimer E., Kalayci T., Schumann S., Suer I., Greco S., Schanze D., Schmeisser M.J., Kuhl S.J., Zenker M. (2022). Functional characterization of a novel TP53RK mutation identified in a family with Galloway-Mowat syndrome. Hum. Mutat..

[81] Thiaville P.C., Iwata-Reuyl D., de Crecy-Lagard V. (2014). Diversity of the biosynthesis pathway for threonylcarbamoyladenosine (t(6)A), a universal modification of tRNA. RNA Biol..

[82] Lin C.A., Ellis S.R., True H.L. (2010). The Sua5 protein is essential for normal translational regulation in yeast. Mol. Cell. Biol..

[83] Harris K.A., Jones V., Bilbille Y., Swairjo M.A., Agris P.F. (2011). YrdC exhibits properties expected of a subunit for a tRNA threonylcarbamoyl transferase. Rna.

[84] Allali-Hassani A., Campbell T.L., Ho A., Schertzer J.W., Brown E.D. (2004). Probing the active site of YjeE: A vital *Escherichia coli* protein of unknown function. Biochem. J..

[85] Luthra A., Swinehart W., Bayooz S., Phan P., Stec B., Iwata-Reuyl D., Swairjo M.A. (2018). Structure and mechanism of a bacterial t6A biosynthesis system. Nucleic Acids Res..

[86] Galperin M.Y., Koonin E.V. (2010). From complete genome sequence to ‘complete’ understanding?. Trends Biotechnol..

[87] Missoury S., Plancqueel S., Li de la Sierra-Gallay I., Zhang W., Liger D., Durand D., Dammak R., Collinet B., van Tilbeurgh H. (2018). The structure of the TsaB/TsaD/TsaE complex reveals an unexpected mechanism for the bacterial t6A tRNA-modification. Nucleic Acids Res..

[88] Bork P., Sander C., Valencia A. (1992). An ATPase domain common to prokaryotic cell cycle proteins, sugar kinases, actin, and hsp70 heat shock proteins. Proc. Natl. Acad. Sci. USA.

[89] Buss K.A., Cooper D.R., Ingram-Smith C., Ferry J.G., Sanders D.A., Hasson M.S. (2001). Urkinase: Structure of acetate kinase, a member of the ASKHA superfamily of phosphotransferases. J. Bacteriol..

[90] Hecker A., Graille M., Madec E., Gadelle D., Le Cam E., van Tilbergh H., Forterre P. (2009). The universal Kae1 protein and the associated Bud32 kinase (PRPK), a mysterious protein couple probably essential for genome maintenance in Archaea and Eukarya. Biochem. Soc. Trans..

[91] Harding M.M., Nowicki M.W., Walkinshaw M.D. (2010). Metals in protein structures: A review of their principal features. Crystallogr. Rev..

[92] Shomura Y., Higuchi Y. (2012). Structural basis for the reaction mechanism of S-carbamoylation of HypE by HypF in the maturation of [NiFe]-hydrogenases. J. Biol. Chem..

[93] Handford J.I., Ize B., Buchanan G., Butland G.P., Greenblatt J., Emili A., Palmer T. (2009). Conserved network of proteins essential for bacterial viability. J. Bacteriol..

[94] Msadek T. (2009). Grasping at shadows: Revealing the elusive nature of essential genes. J. Bacteriol..

[95] Aydin I., Saijo-Hamano Y., Namba K., Thomas C., Roujeinikova A. (2011). Structural analysis of the essential resuscitation promoting factor YeaZ suggests a mechanism of nucleotide regulation through dimer reorganization. PLoS ONE.

[96] Nichols C.E., Johnson C., Lockyer M., Charles I.G., Lamb H.K., Hawkins A.R., Stammers D.K. (2006). Structural characterization of *Salmonella typhimurium* YeaZ, an M22 O-sialoglycoprotein endopeptidase homolog. Proteins.

[97] Xu Q., McMullan D., Jaroszewski L., Krishna S.S., Elsliger M.A., Yeh A.P., Abdubek P., Astakhova T., Axelrod H.L., Carlton D. (2010). Structure of an essential bacterial protein YeaZ (TM0874) from *Thermotoga maritima* at 2.5 A resolution. Acta Crystallogr. Sect. F Struct. Biol. Commun..

[98] Vecchietti D., Ferrara S., Rusmini R., Macchi R., Milani M., Bertoni G. (2016). Crystal structure of YeaZ from *Pseudomonas aeruginosa*. Biochem. Biophys. Res. Commun..

[99] Hecker A., Lopreiato R., Graille M., Collinet B., Forterre P., Libri D., van Tilbeurgh H. (2008). Structure of the archaeal Kae1/Bud32 fusion protein MJ1130: A model for the eukaryotic EKC/KEOPS subcomplex. EMBO J..

[100] Li J., Ma X., Banerjee S., Chen H., Ma W., Bode A.M., Dong Z. (2021). Crystal structure of the human PRPK-TPRKB complex. Commun. Biol..

[101] Zhang W., Collinet B., Graille M., Daugeron M.C., Lazar N., Libri D., Durand D., van Tilbeurgh H. (2015). Crystal structures of the Gon7/Pcc1 and Bud32/Cgi121 complexes provide a model for the complete yeast KEOPS complex. Nucleic Acids Res..

[102] Teplyakov A., Obmolova G., Tordova M., Thanki N., Bonander N., Eisenstein E., Howard A.J., Gilliland G.L. (2002). Crystal structure of the YjeE protein from *Haemophilus influenzae*: A putative Atpase involved in cell wall synthesis. Proteins.

[103] Brown E.D. (2005). Conserved P-loop GTPases of unknown function in bacteria: An emerging and vital ensemble in bacterial physiology. Biochem. Cell Biol..

[104] Leipe D.D., Wolf Y.I., Koonin E.V., Aravind L. (2002). Classification and evolution of P-loop GTPases and related ATPases. J. Mol. Biol..

[105] Hu Y., Tang H.B., Liu N.N., Tong X.J., Dang W., Duan Y.M., Fu X.H., Zhang Y., Peng J., Meng F.L. (2013). Telomerase-null survivor screening identifies novel telomere recombination regulators. PLoS Genet..

[106] He M.H., Liu J.C., Lu Y.S., Wu Z.J., Liu Y.Y., Wu Z., Peng J., Zhou J.Q. (2019). KEOPS complex promotes homologous recombination via DNA resection. Nucleic Acids Res..

[107] Peng J., He M.H., Duan Y.M., Liu Y.T., Zhou J.Q. (2015). Inhibition of telomere recombination by inactivation of KEOPS subunit Cgi121 promotes cell longevity. PLoS Genet..

[108] Daugeron M.C., Lenstra T.L., Frizzarin M., El Yacoubi B., Liu X., Baudin-Baillieu A., Lijnzaad P., Decourty L., Saveanu C., Jacquier A. (2011). Gcn4 misregulation reveals a direct role for the evolutionary conserved EKC/KEOPS in the t6A modification of tRNAs. Nucleic Acids Res..

[109] Liu Y.Y., He M.H., Liu J.C., Lu Y.S., Peng J., Zhou J.Q. (2018). Yeast KEOPS complex regulates telomere length independently of its t(6)A modification function. J. Genet. Genom..

[110] Rojas-Benitez D., Eggers C., Glavic A. (2017). Modulation of the Proteostasis Machinery to Overcome Stress Caused by Diminished Levels of t6A-Modified tRNAs in Drosophila. Biomolecules.

[111] Hyun H.S., Kim S.H., Park E., Cho M.H., Kang H.G., Lee H.S., Miyake N., Matsumoto N., Tsukaguchi H., Cheong H.I. (2018). A familial case of Galloway-Mowat syndrome due to a novel TP53RK mutation: A case report. BMC Med. Genet..

[112] Ferreira-Cerca S., Sagar V., Schafer T., Diop M., Wesseling A.M., Lu H., Chai E., Hurt E., LaRonde-LeBlanc N. (2012). ATPase-dependent role of the atypical kinase Rio2 on the evolving pre-40S ribosomal subunit. Nat. Struct. Mol. Biol..

[113] Lee K.T., Hong J., Lee D.G., Lee M., Cha S., Lim Y.G., Jung K.W., Hwangbo A., Lee Y., Yu S.J. (2020). Fungal kinases and transcription factors regulating brain infection in *Cryptococcus neoformans*. Nat. Commun..

[114] Uhlen M., Fagerberg L., Hallstrom B.M., Lindskog C., Oksvold P., Mardinoglu A., Sivertsson A., Kampf C., Sjostedt E., Asplund A. (2015). Tissue-based map of the human proteome. Science.

[115] Karlsson M., Zhang C., Mear L., Zhong W., Digre A., Katona B., Sjostedt E., Butler L., Odeberg J., Dusart P. (2021). A single-cell type transcriptomics map of human tissues. Sci. Adv..

[116] Beenstock J., Sicheri F. (2021). The structural and functional workings of KEOPS. Nucleic Acids Res..

[117] Morin A., Auxilien S., Senger B., Tewari R., Grosjean H. (1998). Structural requirements for enzymatic formation of threonylcarbamoyladenosine (t6A) in tRNA: An in vivo study with *Xenopus laevis* oocytes. RNA.

[118] Karst J.C., Foucher A.E., Campbell T.L., Di Guilmi A.M., Stroebel D., Mangat C.S., Brown E.D., Jault J.M. (2009). The ATPase activity of an ‘essential’ *Bacillus subtilis* enzyme, YdiB, is required for its cellular function and is modulated by oligomerization. Microbiology.

[119] Arroyo M.N., Green J.A., Cnop M., Igoillo-Esteve M. (2021). tRNA Biology in the Pathogenesis of Diabetes: Role of Genetic and Environmental Factors. Int. J. Mol. Sci..

[120] Suzuki T., Suzuki T. (2014). A complete landscape of post-transcriptional modifications in mammalian mitochondrial tRNAs. Nucleic Acids Res..

[121] Lipska-Zietkiewicz B.S., Ozaltin F., Holtta T., Bockenhauer D., Berody S., Levtchenko E., Vivarelli M., Webb H., Haffner D., Schaefer F. (2020). Genetic aspects of congenital nephrotic syndrome: A consensus statement from the ERKNet-ESPN inherited glomerulopathy working group. Eur. J. Hum. Genet..

[122] Edvardson S., Prunetti L., Arraf A., Haas D., Bacusmo J.M., Hu J.F., Ta-Shma A., Dedon P.C., de Crecy-Lagard V., Elpeleg O. (2017). tRNA N6-adenosine threonylcarbamoyltransferase defect due to KAE1/TCS3 (OSGEP) mutation manifest by neurodegeneration and renal tubulopathy. Eur. J. Hum. Genet..

[123] Carney E.F. (2017). Nephrotic syndrome: Novel monogenic causes of Galloway-Mowat syndrome. Nat. Rev. Nephrol..

[124] Xu S., Hu L., Yang L., Wu B., Cao Y., Zhang R., Xu X., Ma H., Zhou W., Cheng G. (2022). Galloway-Mowat Syndrome Type 3 Caused by OSGEP Gene Variants: A Case Report and Literature Review. Front. Pediatr..

[125] Suzuki T., Yashiro Y., Kikuchi I., Ishigami Y., Saito H., Matsuzawa I., Okada S., Mito M., Iwasaki S., Ma D. (2020). Complete chemical structures of human mitochondrial tRNAs. Nat. Commun..

[126] Abel M.E., Zhang X., Asah S.M., Wolfinger A., McCullumsmith R.E., O’Donovan S.M. (2021). KEOPS complex expression in the frontal cortex in major depression and schizophrenia. World J. Biol. Psychiatry.

[127] Treimer E., Niedermayer K., Schumann S., Zenker M., Schmeisser M.J., Kuhl S.J. (2021). Galloway-Mowat syndrome: New insights from bioinformatics and expression during *Xenopus* embryogenesis. Gene Expr. Patterns.

[128] Braun D.A., Shril S., Sinha A., Schneider R., Tan W., Ashraf S., Hermle T., Jobst-Schwan T., Widmeier E., Majmundar A.J. (2018). Mutations in WDR4 as a new cause of Galloway-Mowat syndrome. Am. J. Med. Genet. A.

[129] Alexandrov A., Martzen M.R., Phizicky E.M. (2002). Two proteins that form a complex are required for 7-methylguanosine modification of yeast tRNA. RNA.

